# USP14 governs CYP2E1 to promote nonalcoholic fatty liver disease through deubiquitination and stabilization of HSP90AA1

**DOI:** 10.1038/s41419-023-06091-6

**Published:** 2023-08-26

**Authors:** Dongqin Wei, Xin Tian, Longbo Zhu, Han Wang, Chao Sun

**Affiliations:** College of Animal Science and Technology, Northwest A&F University, Yangling, 712100 Shanxi China

**Keywords:** Ubiquitylation, Metabolic syndrome

## Abstract

Nonalcoholic fatty liver disease (NAFLD) begins with excessive triglyceride accumulation in the liver, and overly severe hepatic steatosis progresses to nonalcoholic steatohepatitis (NASH), which is characterized by lipid peroxidation, inflammation, and fibrosis. Ubiquitin-specific proteinase 14 (USP14) regulates inflammation, hepatocellular carcinoma and viral infection, but the effect of USP14 on NAFLD is unknown. The aim of this study was to reveal the role of USP14 in the progression of NAFLD and its underlying mechanism. We demonstrated that hepatic USP14 expression was significantly increased in NAFLD in both humans and mice. Hepatic USP14 overexpression exacerbated diet-induced hepatic steatosis, inflammation and fibrosis in mice, in contrast to the results of hepatic USP14 knockdown. Furthermore, palmitic/oleic acid-induced lipid peroxidation and inflammation in hepatocytes were markedly increased by USP14 overexpression but decreased by USP14 knockdown. Notably, in vivo or in vitro data show that USP14 promotes NAFLD progression in a cytochrome p4502E1 (CYP2E1)-dependent manner, which exacerbates hepatocyte oxidative stress, impairs the mitochondrial respiratory chain and inflammation by promoting CYP2E1 protein levels. Mechanistically, we demonstrated by immunoprecipitation and ubiquitination analysis that USP14 inhibits the degradation of heat shock protein 90 alpha family class A member 1 (HSP90AA1) by decreasing its lysine 48-linkage ubiquitination. Meanwhile, upregulation of HAP90AA1 protein promotes CYP2E1 protein accumulation. Collectively, our data indicate that an unknown USP14-HSP90AA1-CYP2E1 axis contributes to NAFLD progression, and we propose that inhibition of USP14 may be an effective strategy for NASH treatment.

## Introduction

The prevalence of nonalcoholic fatty liver disease (NAFLD) has been rising steeply in recent years, with the current global prevalence in adults increasing by ~25% [1]. NAFLD begins with hepatic lipid accumulation, and significant hepatic steatosis is a risk factor for further disease progression. Nonalcoholic steatohepatitis (NASH) is the more severe stage of NAFLD development and is characterized by excessive hepatic triglyceride (TG) accumulation (steatosis), inflammation, cellular damage, and fibrosis [2]. In more severe cases, cirrhosis and liver cancer may develop. Despite the increasingly serious impact of NAFLD on the world’s health, research on its treatment has been slow, with dietary and lifestyle interventions being the mainstay of treatment, and there are currently no FDA-approved drugs specifically for the disease [3]. Therefore, an investigation into the pathophysiology, regulatory mechanisms, and targets of action between NAFLD and extrahepatic complications will facilitate the development of new drugs.

The pathogenesis of NASH is complex, and two key links cannot be avoided: hepatic steatosis that accompanies insulin resistance (IR) and liver damage and inflammation triggered by oxidative stress [4]. A series of studies have shown that oxidative stress is involved in the whole process of NASH. Notably, the induction of oxidative stress occurs mainly in the mitochondria where cytochrome p4502E1 (CYP2E1) produces excess reactive oxygen species (ROS) [5]. When mitochondrial dysfunction occurs, it leads to the activation of CYP2E1, which directly produces free radicals and ROS that contribute to oxidative stress and lipid peroxidation (LPO), leading to increased fat accumulation, inflammation, and hepatocyte death [6]. The mechanism of in vivo degradation of CYP2E1 has been studied and relies on the ubiquitin‒proteasome system (UPS) [7, 8]. Notably, heat shock protein 90 alpha family class A member 1 (HSP90AA1) is involved in the regulation of the balance between CYP2E1 protein degradation and integrity, and radicicol, an HSP90 inhibitor, results in loss of CYP2E1 activity and protein [9]. This is consistent with a model in which the HSP90-based chaperone machinery is responsible for HSP90-dependent stabilization of CYP2E1 from proteolysis via the UPS [10]. In vitro studies have shown that CYP2E1 interacts with HSP90AA1, leading to the dissociation of CYP2E1 from the membrane and the formation of a CYP2E1-HSP90 complex, a state that does not interact with HSP70 to allow E3 ubiquitin ligase CHIP-dependent proteasomal degradation [9, 11]. However, the specific E3 ubiquitinase and deubiquitinase systems involved in this process have not been revealed and deserve further exploration.

The UPS of ubiquitinated proteins is tightly regulated. One of the major regulatory points is the removal of the ubiquitin chain from the substrate by ubiquitin-specific protease 14 (USP14), which reversibly binds the proteasome and confers the ability to edit and reject the substrate [12]. USP14 is a proteasome-associated deubiquitinating enzyme and contains 494 amino acids in its full length. According to its function, it contains two structural domains, an N-terminal ubiquitin-like (UBL) domain and a C-terminal catalytic USP domain (residues 96–494) [12, 13]. Some of the current studies have shown that USP14 shows a positive correlation with liver disease development by regulating several metabolism-related signaling molecules, including hypoxia-inducible factor [14], cAMP-response element binding protein [15], and fatty acid synthase (FASN) [16], to promote tumor proliferation, survival, and liver IR. Thus, the function of USP14 in hepatic steatosis, especially the target of action of hepatic LPO, is not well studied.

In this study, we found that USP14 showed a positive correlation with NAFLD development. Overexpression of USP14 in vivo and in vitro exacerbated LPO, inflammation, and fibrosis in the liver. In contrast, USP14 deficiency has a protective role in NASH. Mechanistically, USP14 stabilizes HSP90AA1 via deubiquitination of the lysine 48 (K48) linkage, which in turn increases CYP2E1 protein to promote NASH progression. Our results suggest that USP14 is a potential target in the treatment of NAFLD.

## Results

### USP14 is significantly upregulated in the pathogenesis of NAFLD

**Fig. 1:**
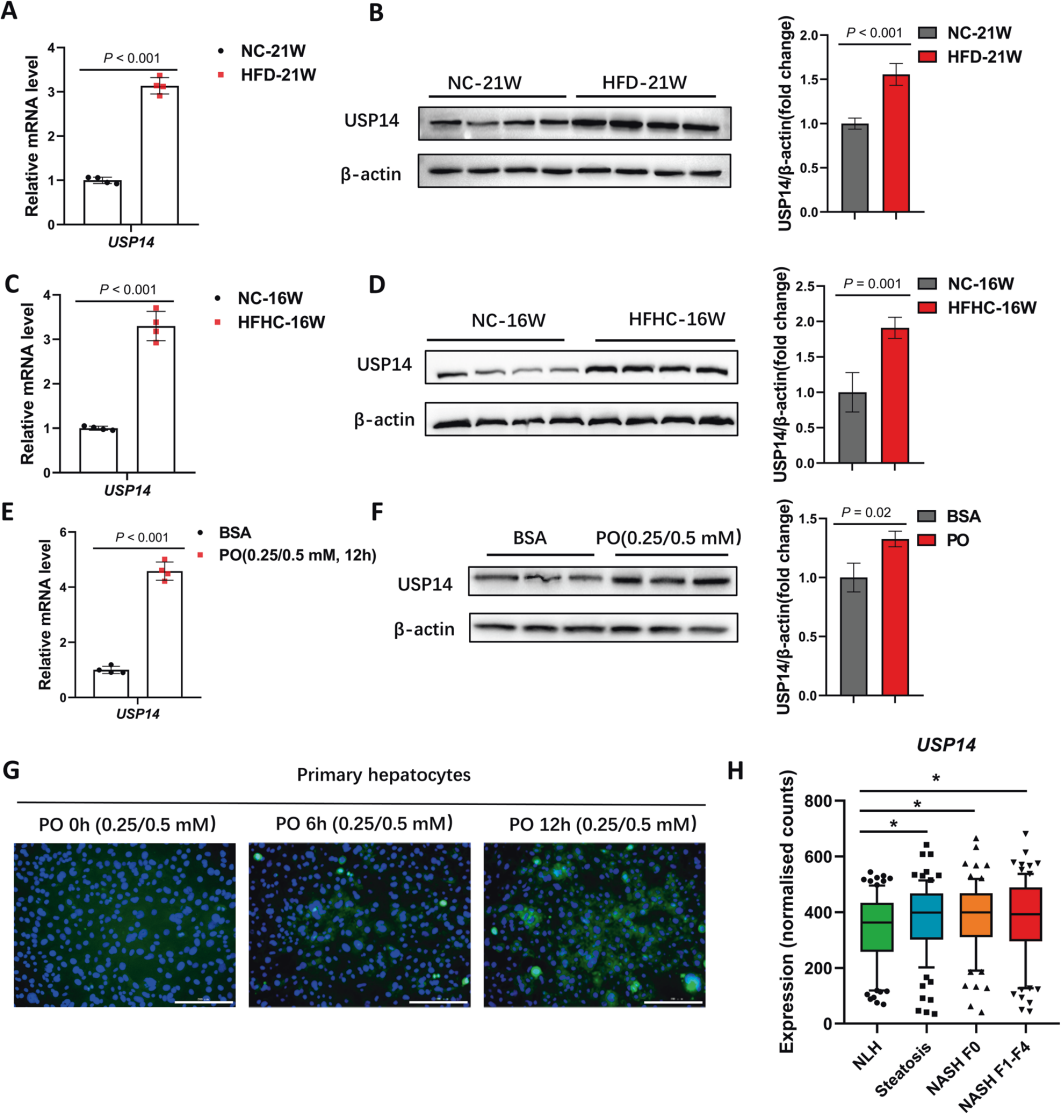
USP14 is upregulated in mouse and human livers as NAFLD progresses. **A**, **B** Quantitative PCR and western blot analysis of USP14 mRNA and protein levels in liver tissue from HFD-induced 21-week mice. **C**, **D** HFHC induced mRNA and protein levels of USP14 in the liver of 16-week mice. **E**, **F** mRNA and protein levels of USP14 in primary hepatocytes treated with PO for 12 h. **G** Immunofluorescence staining to assess USP14 expression in primary mouse hepatocytes treated with PO for the indicated times. Scale bar, 200 μm. **H** Analysis of USP14 expression using RNA-seq data from liver tissue of NASH patients or normal human. * *P* < 0.05 between the two groups.

To investigate the role of USP14 in NAFLD, we examined USP14 expression in the livers of mice fed a high-fat diet (HFD) or high-fat high-cholesterol diet (HFHC). The results showed that HFD or HFHC induced an increase in liver steatosis (Fig. S1A–D). At this time, the mRNA and protein expression of USP14 was significantly increased compared to that in normal (NC-fed) mice (Fig. 1A–D). In vitro experiments showed that USP14 expression was significantly increased in primary mouse hepatocytes treated with palmitic acid (PA, 0.25 mM) and oleic acid (OA, 0.5 mM) (referred to as PO) for 12 h (Fig. 1E–G; Fig. S1E–H). In addition, to explore the potential role of USP14 in the progression of human NAFLD, we downloaded RNA-seq data (GSE163211) from the GEO database for normal liver histology (NLH, *n* = 76), steatosis only (steatosis, *n* = 88), nonalcoholic steatohepatitis without fibrosis (NASH F0, *n* = 72), and NASH with fibrosis stages 1–4 (NASH F1-F4, *n* = 82). Consistent with the results in mice, USP14 expression was significantly upregulated in human NASH samples (Fig. 1H). In conclusion, these data suggest that USP14 is highly expressed in NAFLD and positively correlates with the development of NASH.

### USP14 promotes NAFLD progression

**Fig. 2:**
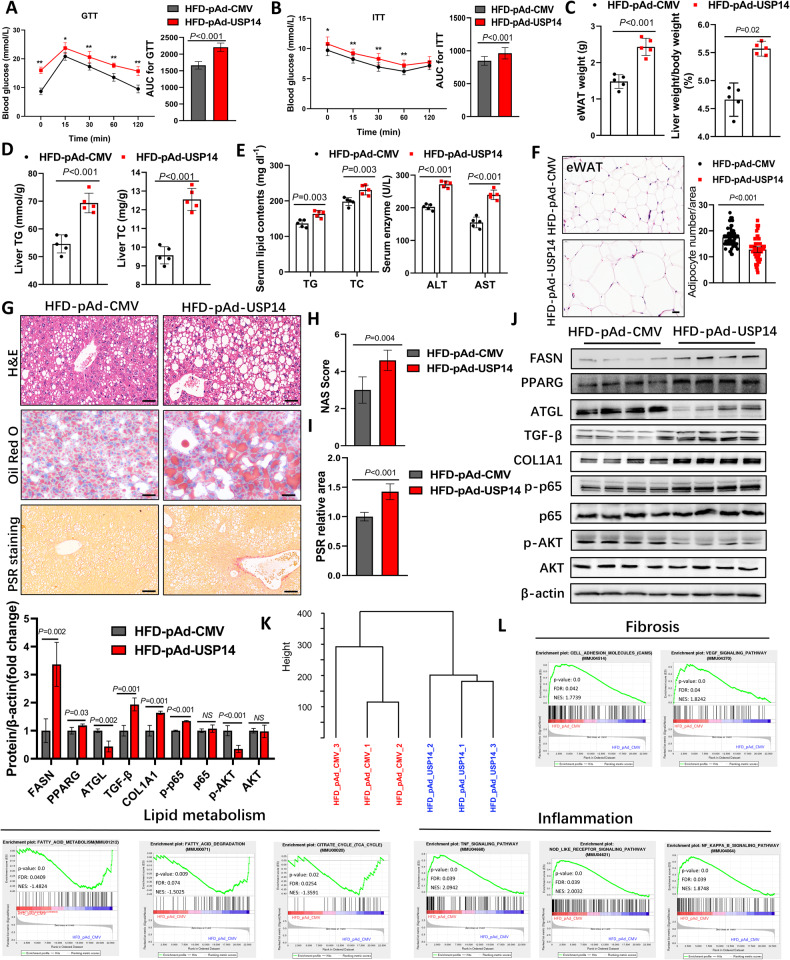
USP14 promotes the progression of NAFLD. **A**, **B** GTT and ITT were performed on 1 week before the end of the experiment (*n* = 12). AUC, area under the curve. **C** eWAT weight and LW/BW ratios in mice 6 weeks after pAd-CMV or pAd-USP14 adenovirus injection (*n* = 5). **D**, **E** Liver TG, and TC levels and serum TG, TC, AST, and ALT levels in the USP14 overexpression and control groups (*n* = 5). **F** H&E staining and adipocyte counts of eWAT (*n* = 5). **G** Representative images of H&E staining, Oil Red O staining, and PSR staining of livers from USP14-overexpressing and control mice (*n* = 5). Scale bar, 50 μm. **H**, **I** NAS scores and relative quantification of PSR area in histological staining of livers from USP14-overexpressing and control mice. **J** Representative immunoblotting analysis of lipid metabolism-related proteins (FASN, PPARG, and ATGL), inflammation-related proteins (p65 and p-p65), fibrosis-related proteins (TGF-β and COL1A1), and insulin sensitivity proteins (AKT and p-AKT) in liver tissue from USP14-overexpressing and control mice. **K** Hierarchical clustering analysis exhibited the global sample distribution profiles in indicated samples. **L** Gene set enrichment analysis showing activation of pathways associated with lipid metabolism, inflammation, and fibrosis. * *P* < 0.05 for the HFD-pAd-CMV compared to the HFD-pAd-USP14 group; ** *P* < 0.01 for the HFD-pAd-CMV compared to the HFD-pAd-USP14 group. NS, not significant.

To further explore the potential role of USP14 in NAFLD, we investigated the effect of USP14 overexpression on the development of NAFLD. We fed C57BL/6 mice an NC diet and an HFD for 16 weeks and injected adenovirus containing pAd-CMV and pAd-USP14 into the mice (Fig. S2A). Compared to the NC group, the HFD-fed 16-week mice were heavier and developed IR (Fig. S2B–D). Furthermore, 6 weeks after adenovirus injection, Western blotting confirmed USP14 overexpression in the liver (Fig. S2E, F). Overexpression of USP14 promoted HFD-induced epididymal white adipose tissue (eWAT) and body weight gain and glucose intolerance and further increased liver weight-to-body weight (LW/BW) ratios, liver and serum lipid content, and liver alanine aminotransferase (ALT) and aspartate aminotransferase (AST) activities (Fig. 2A–E; Fig. S2G), but food intake did not differ significantly (Fig. S2H). Consistent with these results, histopathological staining showed that under HFD induction, the pAd-USP14 group had a lower number of eWAT adipocytes and more severe hepatic steatosis and fibrosis than in control group (Fig. 2F–I). In addition, USP14 overexpression increased the expression of lipid synthesis-, inflammation-, fibrosis-related proteins (FASN, PPARG, NF-κB, TGF-β, COL1A1) (Fig. 2J; Fig. S2I–J) and decreased the expression of lipolysis- and insulin sensitivity-related protein (ATGL, p-AKT) (Fig. 2J).

To further assess the role of USP14 in the pathogenesis of NAFLD, we performed RNA sequencing analysis of liver tissue from HFD-pAd-USP14 and HFD-pAd-CMV mice (Fig. 2K; Fig. S2K). Gene set enrichment analysis showed that lipid degradation-related signaling pathways were significantly inhibited in the HFD-pAd-USP14 group, while inflammation- and fibrosis-related signaling pathways were activated (Fig. 2L). Heatmaps revealed the activation of lipid metabolism, inflammation, and fibrosis genes in each group (Fig. S2L). These results suggest that overexpression of USP14 exacerbates hepatic steatosis and liver injury in the progression of NAFLD.

### USP14 knockdown alleviates diet-induced NASH

**Fig. 3:**
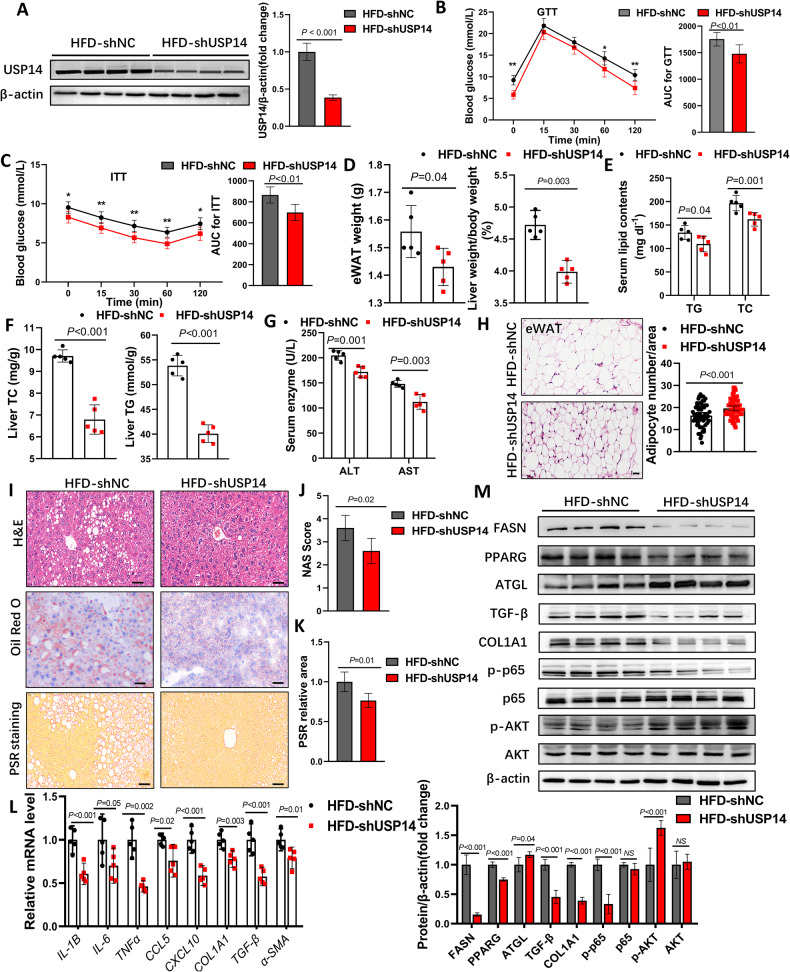
Knockdown of hepatic USP14 alleviates the HFD-induced NAFLD progression. **A** USP14 protein levels in livers of shNC or shUSP14 adenovirus-treated mice. **B**, **C** GTT and ITT analysis of mice 5 weeks after shNC or shUSP14 adenovirus injection (*n* = 12). AUC, area under the curve. **D** Effect of shNC or shUSP14 adenovirus injection on eWAT weight and LW/BW ratios in mice (*n* = 5). **E**–**G** Effect of shNC or shUSP14 adenovirus injection on serum or liver TG and TC levels and serum AST and ALT levels in mice (*n* = 5). **H** H&E staining and adipocyte counts of eWAT (*n* = 5). **I** H&E staining, oil red O staining, and PSR staining from the livers of mice in each group (*n* = 5). Scale bar, 50 μm. **J**, **K** NAS score and relative PSR area from each group of mice. **L** Expression of inflammatory and fibrotic genes in the liver of each group of mice (*n* = 5). **M** Expression of lipid metabolism-related proteins, inflammation-related proteins, fibrosis-related proteins, and insulin sensitivity-related proteins in the liver of mice in each group. * *P* < 0.05 for the HFD-shNC compared to the HFD-shUSP14 group; ** *P* < 0.01 for the HFD-shNC compared to the HFD- shUSP14 group. NS not significant.

To further investigate the function of USP14 in hepatic steatosis, we performed adenoviral shRNA-mediated USP14 knockdown in HFD-treated C57BL/6 mice (Fig. S3A). Adenovirus injection for 6 weeks showed a significant reduction in hepatic USP14 expression in mice (Fig. 3A; Fig. S3B). Mice in the HFD-shUSP14 group exhibited significantly attenuated HFD-induced IR and metabolic syndrome phenotypes (Fig. 3B–D). Systematic reductions in NAFLD-related indicators, including TG and total cholesterol (TC) levels, AST and ALT activities, lipid droplet size, inflammatory infiltration, and fibrosis, were observed in shUSP14 mice compared to the HFD-shNC group (Fig. 3E–K). However, the number of eWAT adipocytes was significantly increased (Fig. 3H). Further qPCR and western blot analysis showed that USP14 knockdown reduced lipid synthesis-, inflammation- and fibrosis-related protein expression and promoted lipolysis- and insulin sensitivity-related protein expression (Fig. 3L, M).

We further evaluated the role of USP14 in the HFHC diet-induced NASH model, which exhibited a more severe inflammatory response and liver fibrosis than the HFD model [17]. Consistent with the phenotype in the HFD model, the knockdown of USP14 alleviated HFHC diet-induced glucose intolerance and reduced liver weight, lipid composition, and liver injury-associated enzyme activities (Fig. S3D–I). Consistent with these findings, histopathological staining showed that hepatic steatosis, injury, and fibrosis were significantly attenuated in USP14 knockdown mice under HFHC stress (Fig. S3J–M). In addition, USP14 knockdown downregulated the protein levels of representative genes associated with inflammation and fibrosis (Fig. S3N), while promoting activation of the insulin signaling pathway (AKT/p-AKT) (Fig. S3N). These results suggest that USP14 knockdown alleviated diet-induced NASH progression.

### USP14 promotes hepatic LPO and inflammation by increasing CYP2E1

**Fig. 4:**
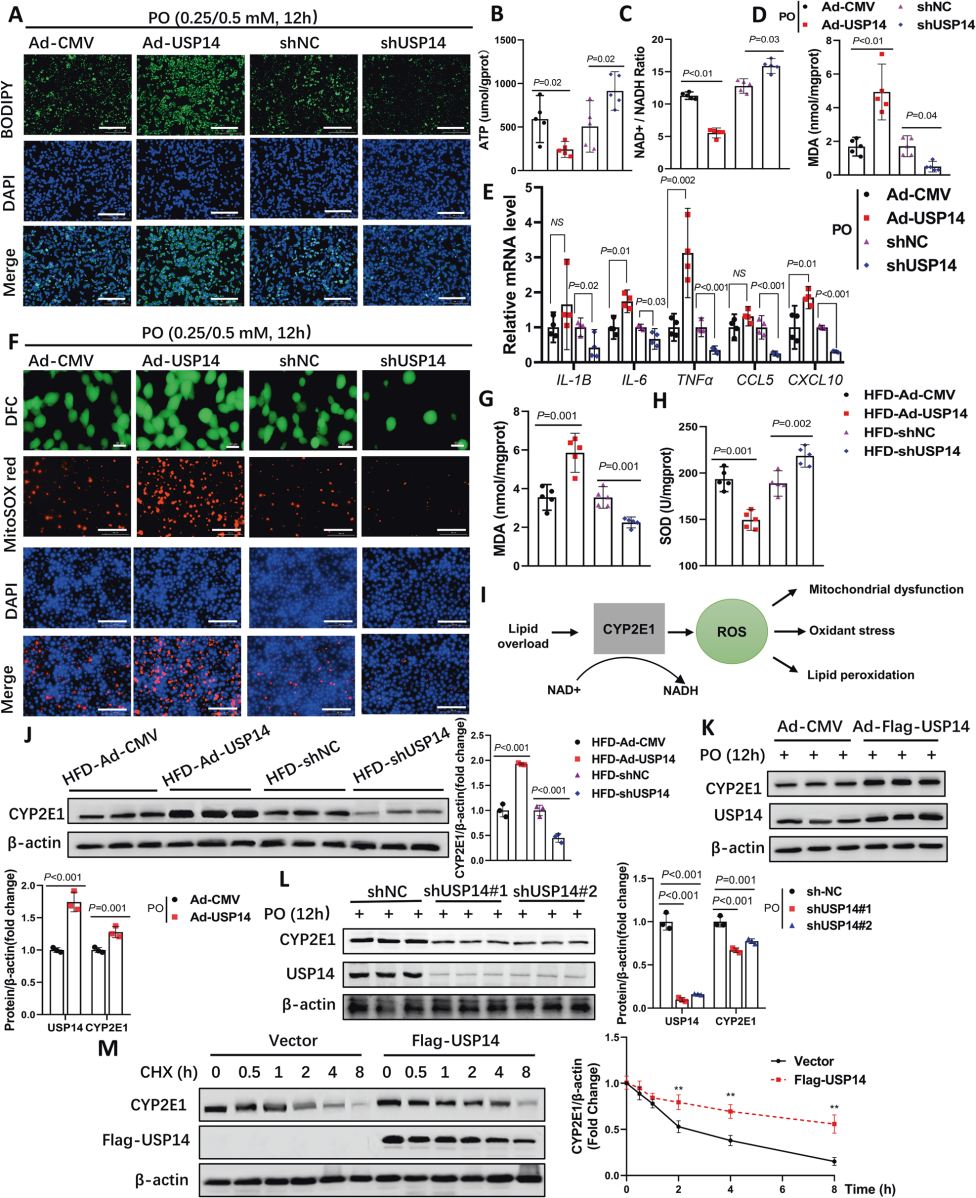
USP14 induces LPO and inflammation by promoting CYP2E1 protein levels. **A** AML12 cells were PO-treated and transfected with the corresponding adenovirus for BODIPY staining (*n* = 3), scale bar, 200 μm. **B**–**D** ATP, NAD^+^/NADH, and MDA levels in AML12 cells treated with PO and transfected with the corresponding adenovirus. **E** Quantitative PCR analysis of the expression of inflammatory genes in each group of cells. **F** Assessment of ROS and mitochondrial ROS in AML12 cells (*n* = 3), ROS scale bar, 50 μm; other scale bar, 100 μm. **G**, **H** Liver MDA and SOD levels in mice after HFD induction and injection of the corresponding adenovirus. **I** Schematic representation of the function of CYP2E1. **J** protein expression of CYP2E1 from the liver of each group of mice. **K**, **L** USP14 and CYP2E1 protein expression in AML12 cells after being treated with PO and transfected with the corresponding vectors. **M** CYP2E1 protein levels of AML12 cells treated with 100 μg/mL cycloheximide for the corresponding time and transfected with Flag-USP14 plasmids or empty vectors. **P < 0.01 for the vector group compared to the Flag-USP14 group. NS not significant.

Numerous studies have shown that during NAFLD, steatosis in the liver induces oxidative stress and further promotes the development of inflammation [18, 19]. On the other hand, inflammation itself promotes ROS production due to increased ROS oxidase activity (NADH) and mitochondrial dysfunction [20, 21]. To investigate whether USP14-induced liver inflammation and fibrosis are related to oxidative stress, PO-treated AML12 cells were transfected with USP14 overexpression and interference vectors (Fig. S4A, B). The results showed that USP14 significantly promoted PO treatment-induced lipid accumulation in hepatocytes (Fig. 4A). Meanwhile, USP14 promoted the reduction in mitochondrial membrane potential and ATP content (Fig. 4B, C; Fig. S4C) and suppressed mitochondrial function-related gene expression (Fig. S4D), which led to the production of malondialdehyde (MDA) (Fig. 4D) and induced inflammatory gene expression in hepatocytes (Fig. 4E). The ROS fluorescence probe showed that PO-induced ROS (DFC) and mitochondrial ROS (MitoSOX) were higher in the Ad-USP14 group (Fig. 4F). In contrast, knockdown of USP14 reversed these phenotypes (Fig. 4A–F). Furthermore, we examined MDA and superoxide dismutase (SOD) levels in the liver. Compared to the HFD-pAd-CMV group, liver MDA levels were significantly higher and SOD levels were significantly lower in pAd-USP14 mice (Fig. 4G–H). These data suggest that USP14 promotes hepatic steatosis in close association with hepatic oxidative stress.

In fact, a potential cause of excess hepatic ROS production is the accumulation of CYP2E1. Previous studies have shown that inhibition of CYP2E1 effectively blocked hepatic ROS production with LPO, reduced liver injury, and inhibited NAFLD progression (Fig. 4I) [4–6, 22]. Therefore, increased CYP2E1 expression is considered to be a marker for early diagnosis of NASH. To further investigate the rationale for the role of USP14 in promoting hepatic steatosis, we assessed the effect of USP14 on CYP2E1 both in vivo and in vitro. Interestingly, overexpression of USP14 did not alter the mRNA levels of CYP2E1 in mouse liver (Fig. S4E) but instead significantly increased CYP2E1 protein levels (Fig. 4J). Consistent with this, liver CYP2E1 protein levels were significantly reduced in USP14 knockdown mice (Fig. 4J). The trend of CYP2E1 expression in cells was consistent with that in vivo (Fig. 4K, L; Fig. S4F). This suggests that USP14 may regulate CYP2E1 protein degradation. To test this possibility, we assessed the half-life of CYP2E1 in AML12 cells transfected with Flag-USP14 or empty vectors and found that the expression of USP14 prolonged the half-life of CYP2E1 (Fig. 4M). These data suggest that USP14 induces inflammation by increasing CYP2E1 protein levels to promote hepatic LPO.

### USP14 governs CYP2E1 by promoting HSP90AA1 protein levels

**Fig. 5:**
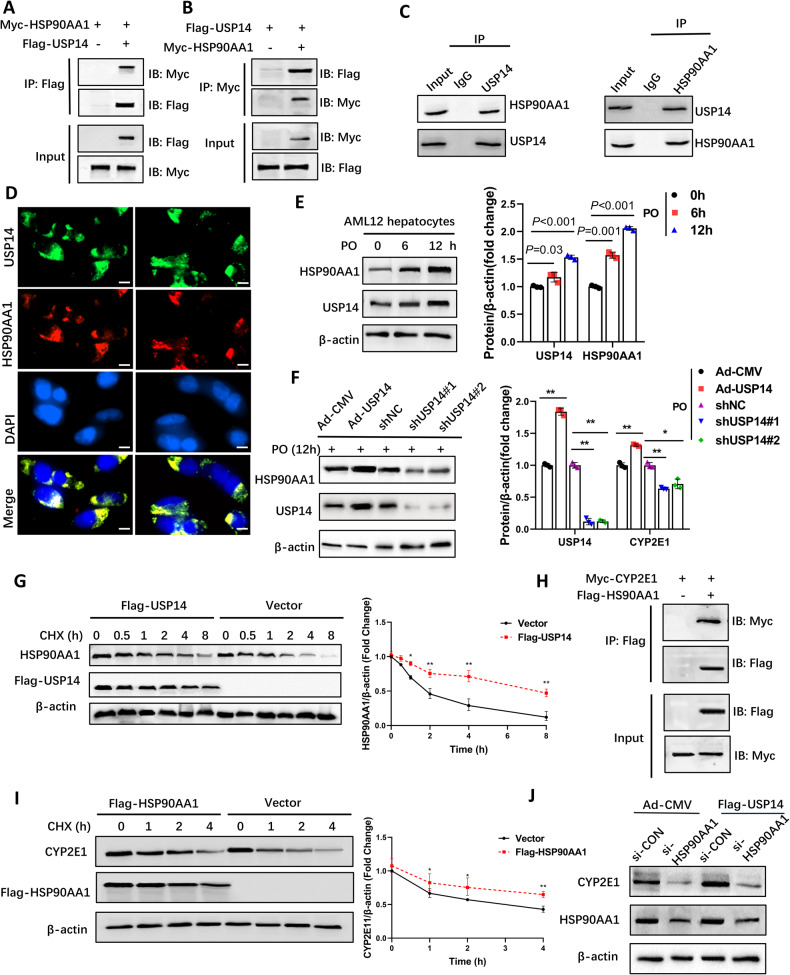
USP14 increases CYP2E1 by promoting HSP90AA1 protein levels. **A**, **B** Exogenous co-IP assays were performed to assess the interaction between USP14 or HSP90AA1 in HEK-293T cells transfected with the corresponding labeled USP14 or HSP90AA1 plasmids. **C** Representative endogenous co-IP analyses show the binding of USP14 to HSP90AA1 in PO-treated AML12 cells for 12 h. IgG was used as the control. **D** Immunofluorescence analysis showing co-localization of USP14 and HSP90AA1 in HEK-293T cells transfected with Flag-USP14 and MYC-HSP90AA1, scale bar, 50 μm. **E** PO treatment of protein levels of USP14 and HSP90AA1 in AML12 cells. **F** HAP90AA1 protein levels in hepatocytes after USP14 overexpression and knockdown. **G** Protein levels of HSP90AA1 in AML12 cells treated with 100 μg/mL cycloheximide for the corresponding time and transfected with Flag-USP14 plasmids or empty vectors. **H** Exogenous co-IP assays were performed to assess the interaction between HSP90AA1 or CYP2E1 in HEK-293T cells transfected with the corresponding labeled HSP90AA1 or CYP2E1 plasmids. **I** Protein levels of CYP2E1 in AML12 cells treated with 100 μg/mL cycloheximide for the corresponding time and transfected with Flag-HSP90AA1 plasmids or empty vectors. **J** Effect of AML12 cells cotransfected with Flag-USP14 and HSP90AA1 siRNA on CYP2E1 protein levels. *P < 0.05 between the two groups. **P < 0.01 between the two groups.

To further investigate whether USP14 interacts with CYP2E1 to promote hepatic LPO, we performed exogenous IP and ubiquitination analyses in AML12 cells. The results showed that USP14 did not interact directly with CYP2E1 to alter its ubiquitination (Fig. S5A–B), suggesting that USP14 may indirectly regulate CYP2E1 protein levels. Liu et al. performed comprehensive proteomic, ubiquitinomic, and interactomic (IP-MS) analyses in USP14 knockdown cells, identifying multiple substrates that bind USP14 [16]. Among the candidates with binding potential, we identified HSP90AA1, which has been reported in many studies to regulate CYP2E1 activity and stability [9, 11]. Our predicted interactions on the GeneMANIA website (http://genemania.org) suggested that HSP90AA1 could be a potential binding partner mediating the regulation between USP14 and CYP2E1 (Fig. S5C). Further endogenous and exogenous IP assays validated the strong interaction between USP14 and HSP90AA1 (Fig. 5A–C). The immunofluorescence results showed that USP14 and HSP90AA1 colocalized (Fig. 5D). Considering the deubiquitinating activity of USP14, we hypothesized that USP14 regulates the stability of HSP90AA1. We examined the correlation between USP14 and HSP90AA1 protein levels. In AML12 cells, HAP90AA1 protein expression gradually increased with increasing USP14 under PO stress (Fig. 5E). Similar results were obtained in USP14-overexpressing and USP14-knockdown hepatocytes and mouse livers (Fig. 5F; Fig. S5D). The results of cycloheximide treatment showed that USP14 prolonged the HSP90AA1 half-life (Fig. 5G). These results suggest that USP14 interacts directly with HSP90AA1 and increases the stability of HSP90AA1.

Furthermore, we verified the regulatory effect of HSP90AA1 on CYP2E1. Co-IP results showed that HSP90AA1 was able to interact directly with CYP2E1 (Fig. 5H) and that the presence of HSP90AA1 significantly prolonged the half-life of CYP2E1 (Fig. 5I). Interference with HSP90AA1 in USP14-overexpressing hepatocytes partially reversed the USP14-induced increase in CYP2E1 expression (Fig. 5J). The above data suggest that USP14 governs CYP2E1 by promoting the protein level of HSP90AA1.

### USP14 reduces K48-linked ubiquitination of HSP90AA1 to stabilize it

**Fig. 6:**
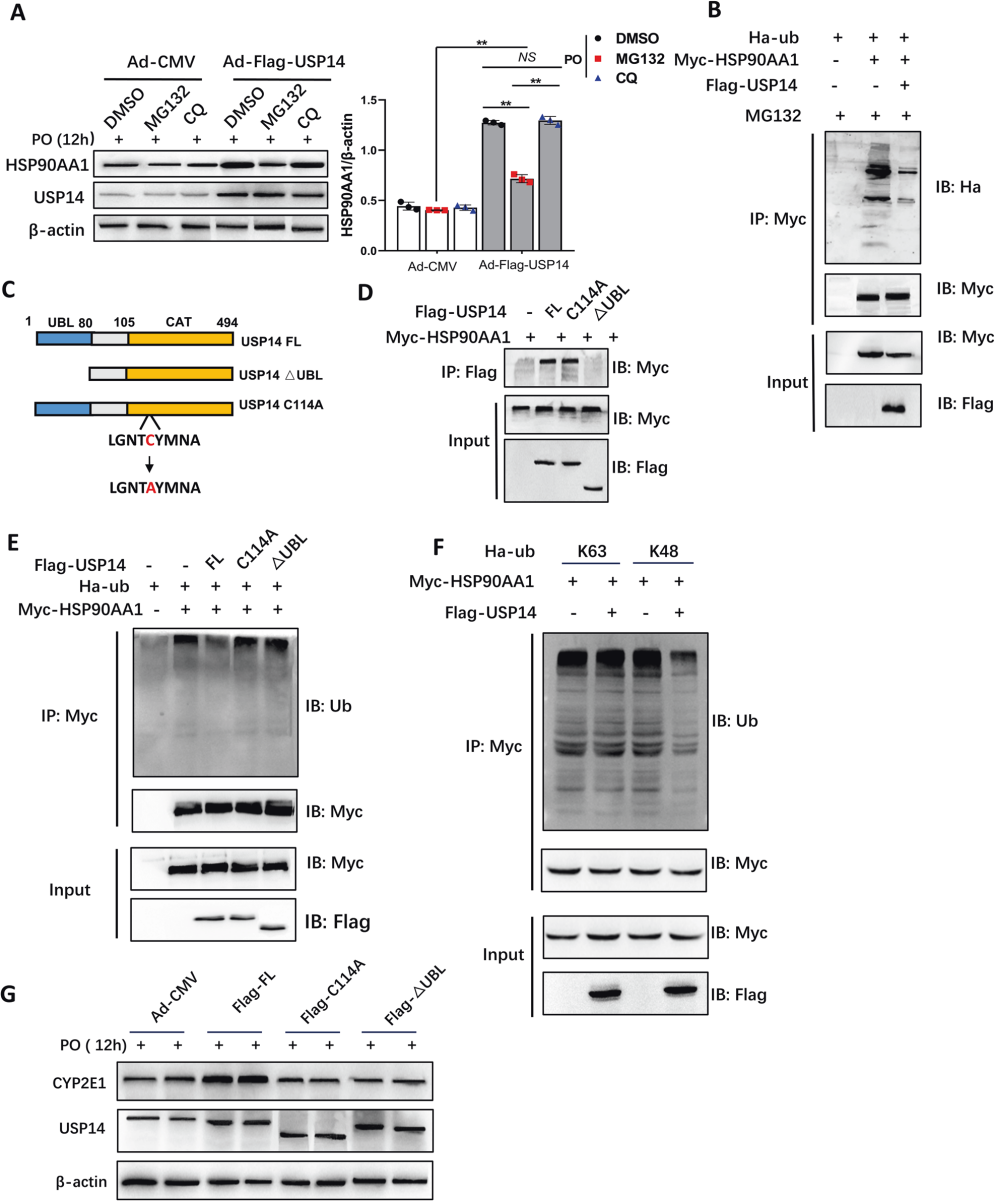
USP14 reduces K48-linked ubiquitination of HSP90AA1 to stabilize it. **A** USP14 and CYP2E1 protein expression in AML12 cells treated with MG132 and CQ and transfected with the corresponding vectors. **B** Co-IP assay to assess ubiquitination of HSP90AA1 in HEK-293T cells transfected with the corresponding labeled USP14, HSP90AA1, or ub plasmids. **C** Construction of an active site mutant of USP14 (USP14 C114A) and a truncation mutant of USP14 lacking the UBL. **D** Co-IP analysis of the interaction domain of USP14 and HAP90AA1. **E** Co-IP analysis of the interaction domain of USP14 on HAP90AA1 deubiquitination. **F** Identification of the type of HAP90AA1 deubiquitination by USP14. **G** Analysis of protein levels of CYP2E1 after AML12 cells were transfected with Ad-CMV, Flag-USP14 FL, Flag-C114A, and Flag-∆UBL. **P < 0.01 between the two groups. NS not significant.

Previous studies have shown that HSP90AA1 is degraded by ubiquitination modifications [23]. Our results suggest that the addition of a proteasome inhibitor (MG132) rather than a lysosomal inhibitor (chloroquine, CQ) can eliminate the accumulation of HSP90AA1 by USP14 (Fig. 6A). Consistent with this, ubiquitination of HSP90AA1 was significantly reduced because of USP14 overexpression (Fig. 6B). We constructed an active site mutant of USP14 (USP14 C114A) and a truncation mutant of USP14 lacking the UBL to determine which structure was responsible for the deubiquitination of HSP90AA1 (Fig. 6C) [24]. The results showed that the ΔUBL truncated mutant was unable to interact with HSP90AA1 (Fig. 6D) and that USP14 FL but not the mutant (C114A and ΔUBL) reduced the ubiquitination of HSP90AA1 (Fig. 6E), indicating that the UBL domain of USP14 was required for HSP90AA1 binding and that CAT and UBL were required to catalyze the deubiquitination of HSP90AA1. Further ubiquitination assays showed that USP14 inhibited K48-linked ubiquitination of HSP90AA1 but not K63-linked ubiquitination (Fig. 6F; Fig. S6A, B). K48-linked ubiquitination has been reported to mediate protein degradation, and our data suggest that USP14 inhibits K48-linked ubiquitination of HSP90AA1 to stabilize it.

We next analyzed the effect of the mutant (C114A and ΔUBL) on CYP2E1 expression. Indeed, mutants of USP14 (C114A and ΔUBL) did not induce an increase in CYP2E1 expression compared to USP14 FL in hepatocytes (Fig. 6G). The above results suggest that USP14 stabilizes CYP2E1 levels by reducing the K48-linked ubiquitination and proteasomal degradation of HSP90AA1.

### The function of USP14 in LPO and inflammation depends on CYP2E1

**Fig. 7:**
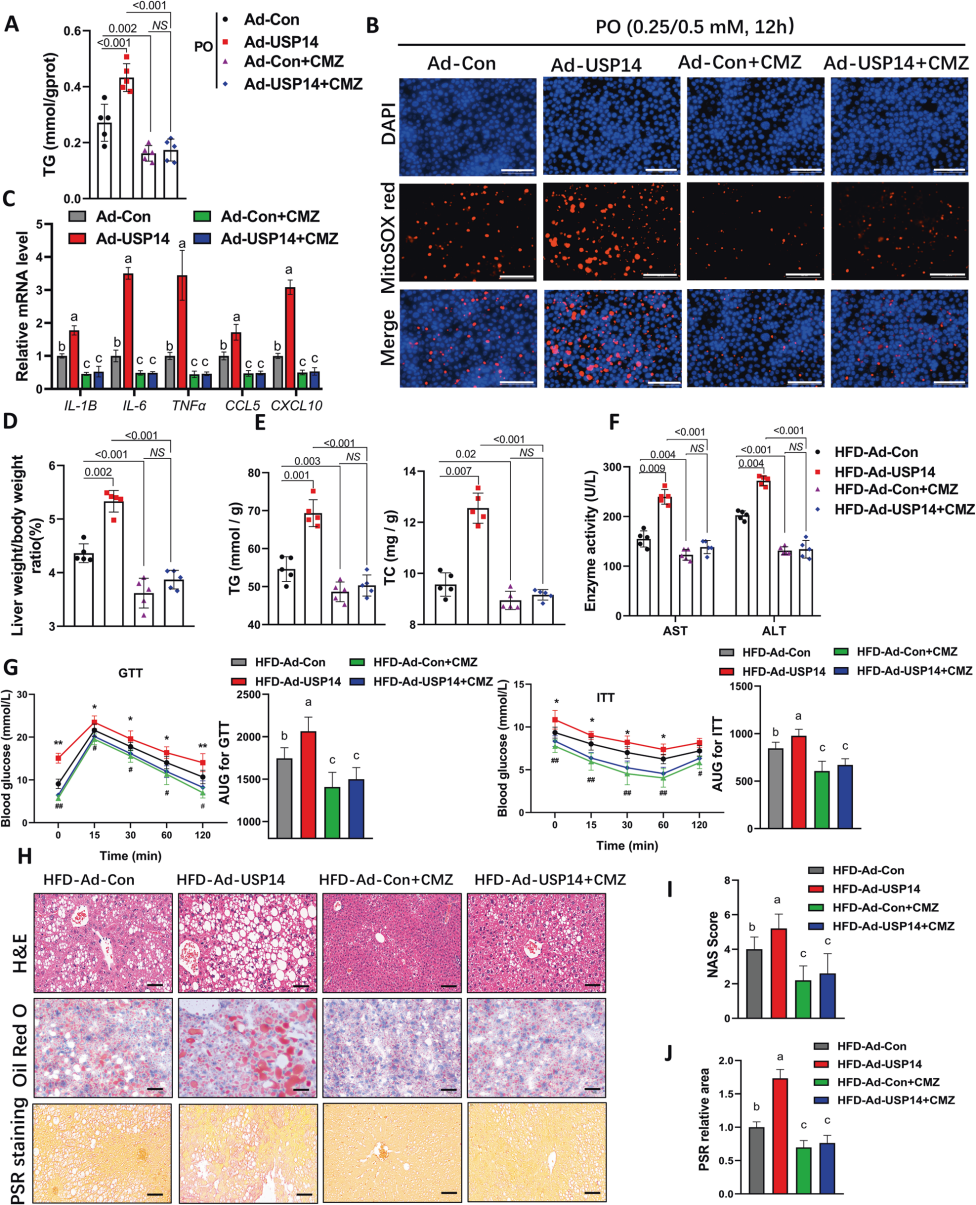
The function of USP14 in LPO and inflammation depends on CYP2E1. **A** Analysis of cellular TG levels in AML12 cells under CMZ treatment and corresponding plasmid transfection. **B** MitoSOX staining of AML12 cells under CMZ treatment and corresponding plasmid transfection (*n* = 3). Scale bar, 100 μm. **C** Expression of inflammation-related genes in AML12 cells under CMZ treatment and corresponding plasmid transfection (*n* = 4). **D**–**F** Effect of CMZ and Ad-USP14 adenovirus injections in mice on LW/BW ratios, liver TG and TC levels, serum AST and ALT levels. **G** GTT and ITT results for each group of mice (*n* = 12). *HFD-Ad-Con vs. HFD-Ad-USP14; ^#^HFD-Ad-Con vs. HFD-Ad-Con+CMZ. *^,#^*P* < 0.05; **^,##^*P* < 0.01. There was no difference between the other groups. **H**–**J** Representative images of H&E staining, Oil Red O staining, and PSR staining of the livers in each group and the corresponding NAS scores and PSR areas. Scale bar, 50 μm. Lowercase letters indicate post hoc analyses significance (*P* < 0.05) and groups with different letters are statistically different per post hoc comparison. NS not significant.

To further investigate whether CYP2E1 activity is required for hepatic USP14 function, we employed a CYP2E1-specific inhibitor, chlormethiazole (CMZ, 40 μM), to treat AML12 cells. As shown in Fig. S7A, CMZ significantly reduced the expression of CYP2E1 in the cells. After PO treatment CMZ mostly reversed the increase in lipid accumulation, oxidative stress, and inflammatory gene expression caused by USP14 overexpression (Fig. 7A–C).

To further assess whether the function of USP14 is dependent on CYP2E1 in vivo, we administered CMZ (50 mg/kg BW) and pAd-USP14 adenovirus to HFD-induced C57BL/6 mice. The immunoblotting results confirmed that CYP2E1 expression was reduced in the liver, while the protein expression of USP14 was increased (Fig. S7B). Consistent with the in vitro results, the Ad-USP14 + CMZ group reversed the trend of USP14-mediated increases in LW/BW ratios, lipid levels (TG and TC), and liver injury-related enzyme activities (AST and ALT) (Fig. 7D–F), but there were no significant changes in feed intake and body weight (Fig. S7C, D). In addition, CYP2E1 inhibition alleviated IR and glucose intolerance in the Ad-USP14 group mice (Fig. 7G). Histological and qPCR analyses revealed that CYP2E1 inhibition alleviated hepatic steatosis, inflammation, and fibrosis in USP14-overexpressing mice (Fig. 7H–J; Fig. S7E). The above results suggest that CYP2E1 is required for the function of USP14 in the pathogenesis of NASH.

## Discussion

Dysregulation of hepatic UPS function has been detected at different stages of NAFLD due to various stimuli (e.g., ROS, endoplasmic reticulum stress, toxic lipids) [25, 26]. The main substrates targeted by dysregulated UPS include NF-κB, PPARγ, CYP2E1 and SREBPs, and Wnt/β-linked protein signaling, which induce lipid accumulation, ROS, and inflammation [4, 25, 27, 28]. The USP family is the most versatile and powerful deubiquitinating enzyme that has been studied, and USP14, which belongs to the USP family, is widely involved in different signaling pathways mediating proliferation and metastasis in hepatocellular carcinoma [12]. In NAFLD, Liu et al. were the first to report that USP14 targets FASN and promotes hepatic lipid accumulation [16]. Few subsequent studies have explored the potential correlation between USP14 and NAFLD. Furthermore, studies over the years have established a ‘two-hit’ theory of NASH pathogenesis. In addition to the first stage of hepatic steatosis (i.e., NAFL), a second ‘hit’ from other factors (e.g., oxidative stress) is necessary for the development of NASH [1]. Liu’s study revealed the role of USP14 in the formation of NAFL. Our study establishes the function of USP14 in the second ‘hit’, i.e., to exacerbate mitochondrial damage and ROS formation in the liver and to induce LPO and inflammation. The role of USP14 in NASH is multidimensional. This suggests that USP14 holds promise as a potential therapeutic target in all aspects of NASH development.

Indeed, increased CYP2E1 protein expression and activity have been found in obesity, alcoholic liver disease (ALD), and NAFLD in both humans and rodents. Increased CYP2E1 leads to mitochondrial respiratory chain electron leakage, inducing ROS production and leading to oxidative stress [6]. Oxidant stress is a pathogenic factor for the onset of ALD and NAFLD, which contributes to hepatic LPO with more severe inflammation, fibrosis, and necrosis [6]. Among the cytochrome P450 family, CYP2E1 has been identified as the most relevant for ALD as it is highly inducible and it has high catalytic activity for alcohol [29]. On the other hand, nonalcoholic steatosis also induces CYP2E1 expression and activation, and activated CYP2E1 likewise leads to oxidative stress [22]. This contributes to the progression of fatty liver from steatosis to steatohepatitis. Our study found increased expression of hepatic USP14 with the development of NAFLD, which directly led to an increase of CYP2E1, inducing more severe ROS, mitochondrial damage, and inflammation. In contrast, inhibition of CYP2E1 reversed the hepatic phenotypes in USP14-overexpressing mice. This also suggests that CYP2E1 is a target of USP14 to exacerbate the progression of NAFLD.

Notably, CYP2E1 is degraded via UPS, which involves multisite protein phosphorylation and ubiquitination. Studies have shown that the main specific E3 ubiquitinating enzymes involved in CYP2E1 degradation are gp78 and CHIP [30]. Monitoring of Ub-derived CYP2E1 modification sites by HPLC‒MS/MS identified K84, K87, K275, K410, K420, K422, K428, K434, and K461s as gp78 specific ubiquitination sites in human liver [8]. We found no direct deubiquitination of CYP2E1 by USP14. This may be because USP14 indirectly regulates CYP2E1 protein levels. Due to the role of molecular chaperones in protein folding and degradation, the extensive involvement of HSP90 in regulating CYP2E1 stability [7, 9, 11]. We observed that USP14 inhibited the degradation of HSP90AA1 through deubiquitination, leading to the accumulation of hepatic CYP2E1. Accordingly, we hypothesized that the downstream target of the USP14-HSP90AA1 axis is CYP2E1. In line with our hypothesis, inhibition of CYP2E1 abolishes the role of USP14 in NAFLD. Here, we provided the first evidence that USP14 inhibited HSP90AA1 degradation via post-translational modification. Previous studies have shown that HSP90 targets PPARγ or SREBPs to regulate lipid metabolism in the liver [31, 32]. Although we have proposed a mechanism of action by which the USP14-HSP90AA1 axis regulates NAFLD through CYP2E1. However, considering that HSP90AA1 is widely involved in a variety of cellular processes, whether this axis is involved in other protein interactions remains to be explored.

Ubiquitination-based degradation of certain proteins has become an important direction for drug development. However, a complete cure for NASH with only a single target is unrealistic, and the limitation of this paper is also in the fact that deubiquitinating enzymes that act directly on the UPS degradation of CYP2E1 have not been identified, and other studies have not been reported. This will be the direction of our future efforts. Another limitation in our study is the lack of liver-specific USP14 knockout mice. This is because changes in the metabolic phenotypes of obesity in mice were also observed under our model. The exact role of USP14 in fatty liver could be better clarified if liver-specific USP14 knockout model was used in the future.

**Fig. 8:**
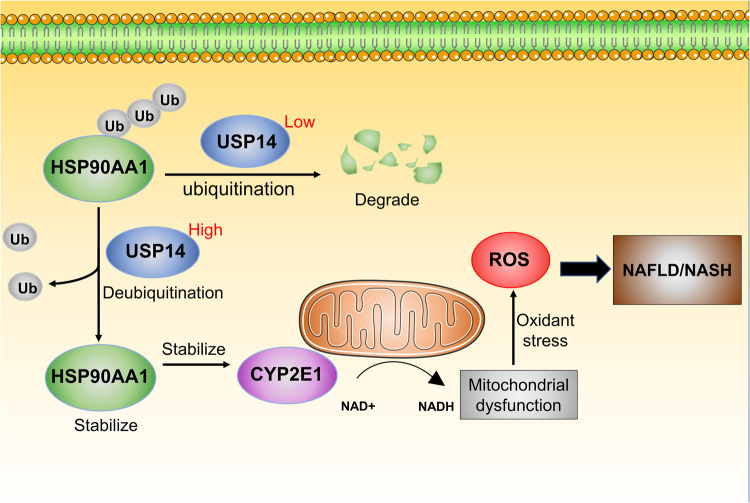
Schematic mechanism illustrating USP14-promoted NAFLD. USP14 stabilises HSP90AA1 by deubiquitination. stabilised HSP90AA1 promotes mitochondrial dysfunction and lipid peroxidation by increasing CYP2E1 proteins in NAFLD . Ub, ubiquitination modification.

In conclusion, our study elucidates that USP14 plays a key role in the pathogenesis of NASH (Fig. 8). More importantly, our data also suggest novel mechanisms and functional links between USP14 and CYP2E1 in the biology of hepatic LPO, providing a new therapeutic target for NASH treatment.

## Materials and methods

### Ethical compliance

All animal experiments were approved by the Northwest A&F University Ethics Committee and followed the principles of the NIH Guide for Care and Use of Laboratory Animals.

### Animal models and treatment

Male C57BL/6 J wild-type mice aged 8 weeks were purchased from the Laboratory Animal Center of the Fourth Military Medical University (Xi’an, China). Mice were reared in a temperature-controlled environment (25 ± 1 °C) with a 12-hour light/dark cycle and free access to water and food throughout the experiment. To model steatosis, mice were fed an HFD (protein, 20%; fat, 60%; carbohydrate, 20%) for 22 weeks or an HFHC (protein, 14%; fat, 42%; carbohydrate, 44%; cholesterol, 0.2%) for 16 weeks. Mice fed a normal chow (NC) diet served as controls. The recombinant adenovirus carrying the mouse USP14 promoter was constructed by the Miaoling Plasmid Platform (Wuhan, China) and named pAd-USP14. The primers used in this study are shown in Table S1. To achieve hepatic USP14 overexpression, mice were injected with pAd-USP14 (1 × 10^9^ PFU) via the tail vein after 16 weeks of HFD feeding, once a week for 6 weeks, and pAd-CMV was injected into control mice. USP14-specific shRNAs were also purchased from the Miaoling Plasmid Platform. The sense target sequence for shUSP14 is 5′-CCTGCTTACTTAACTATTCAA-3′. To silence USP14 expression in the liver, mice treated with HFD or HFHC for 16 weeks were injected with shUSP14 and shCtrl adenoviruses once a week for 6 weeks (1 × 10^8^ PFU). All viruses were purified by the cesium chloride method and dialyzed in PBS containing 10% glycerol before injection.

Furthermore, to explore whether the function of USP14 is dependent on CYP2E1, we administered clomethiazole (CMZ, 50 mg/kg) intraperitoneally to HFD-induced USP14-overexpressing mice or pAd-CMV control mice every other day for four weeks [33]. CMZ was purchased from MedChemExpress (New Jersey, USA).

### Cell culture and treatment

Primary mouse hepatocytes were isolated and cultured according to the method previously described [34] in our laboratory. Primary hepatocytes, AML12 cells, and HEK-293T cells were cultured in a 5% thermostatic CO2 incubator supplemented with 10% fetal bovine serum (FBS, 10100147, Gibco, CA, USA) and 1% penicillin‒streptomycin (15140122, Gibco). To induce NAFLD models in vitro, hepatocytes were treated with medium containing 0.25 mM palmitic acid (PA, P0500, Sigma‒Aldrich, St. Louis, MO, USA) and 0.5 mM oleic acid (OA, 112-80-1, MedChemExpress) for the indicated times, and bovine serum albumin without fatty acids was used as a control. To enhance or interfere with USP14 expression, AML12 cells were infected with adenovirus produced from the above constructs pAd-USP14, pAd-CMV, shUSP14, and shCtrl, and cells were collected to determine the appropriate metrics.

### Mouse experiments

Food intake and body weight data were recorded every other day for all mice. The glucose tolerance test (GTT) and insulin tolerance test (ITT) were performed one week before the end of the test. Briefly, mice were injected intraperitoneally with glucose (1.5 g/kg) or insulin (1 U/kg) after fasting, and then blood glucose was measured at the indicated times (0, 15, 30, 60, and 120 min) after the injection. Serum ALT and AST activities were measured using commercial kits according to the manufacturer’s instructions (BC1555 for ALT and BC1565 for AST, Solarbio, Beijing, China).

### Histological analysis

Mouse livers or eWAT were fixed overnight in 4% paraformaldehyde solution and paraffin-embedded. The embedded samples were cut to 5 µm thickness and then stained with hematoxylin and eosin (H&E) to assess cellular morphological changes in the tissue. Next, paraffin-embedded sections of the liver were stained with picrosirius red (PSR) to assess collagen deposition. Alternatively, liver tissue was embedded at the optimal cutting temperature, cut to 8 µm thickness, and stained using Oil Red O to observe liver lipid accumulation. Finally, stained images were obtained using light microscopy.

### TG and TC measurement

TG and TC were extracted from mouse liver or cells according to methods previously described in our laboratory [34]. In brief, appropriate amounts of samples were homogenized or lysed in a solution of chloroform and methanol (3:2), dried under a stream of nitrogen, and resuspended in isopropanol. The samples were then assayed for TG and TC levels using a commercial kit (MAK040 for TG and MAK043 for TC, Sigma‒Aldrich) according to the instructions. Mouse serum samples were assayed for TG and TC directly according to the kit instructions.

### MDA and SOD analysis

Both MDA and SOD in the liver or cells were analyzed using commercial kits (S0131S for MDA and S0101S for SOD, Beyotime, Shanghai, China). Liver tissues or cells were homogenized or lysed in a solution containing 20 mM Tris (pH 7.5), 150 mM NaCl, and 1% Triton X-100, followed by protein quantification. The homogenate or lysate was further centrifuged at 12,000 × *g* for 10 min to obtain the supernatant for subsequent assays. The analysis was carried out according to the kit instructions.

### Intracellular ATP and NAD+/NADH assays

Intracellular ATP and NAD^+^/NADH levels were measured using the ATP Assay Kit (S0026, Beyotime) and the NAD^+^/NADH Assay Kit (S0175, Beyotime) according to the manufacturer’s instructions.

### Measurement of mitochondrial membrane potential

Mitochondrial membrane potential was measured using the jc-1 probe (ab113850, Abcam, Cambridge, UK). Cells were washed with PBS followed by the addition of JC1 working solution and incubated for 10 min at 37 °C. After three washes, cells were stained with DAPI (C1002, Beyotime) under light-protected conditions to visualize the nuclei. Finally, fluorescence microscopy was used to observe the intensity of the red and green fluorescence signals and to calculate the ratio of the two fluorescence intensities.

### Cellular ROS and mitochondrial ROS assays

To assess total cellular ROS and mitochondrial ROS levels, AML12 cells were stained with 2,7-dichlorodihydrofluorescein diacetate (DCFH-DA, YEASEN, Shanghai, China) and a mitochondrial superoxide indicator (MitoSOX Red, YEASEN), respectively, and incubated at 37 °C for 1 h in the dark. The nuclei were then infiltrated with DAPI, washed with PBS, and then observed by fluorescence microscopy.

### BODIPY staining

After treatment of primary mouse hepatocytes or AML12 cells with PAOA (0.25/0.5 mM) for 12 h, the cells were incubated for 30 min with 10 μM BODIPY 493/503 (216434-81-0, MedChemExpress) working solution and washed three times with PBS and nuclei were stained with DAPI for 3 min. Cells were washed again 3 times with PBS and imaged by fluorescence microscopy.

### Immunofluorescence

HEK-293T cells were cotransfected with Flag-USP14 and Myc-HSP90AA1 plasmids and cells were collected and fixed using 4% paraformaldehyde for 5 min. Cells were permeabilized using 0.25% Triton X-100 and blocked with 1% BSA for 2 h. Cells were sequentially incubated with primary antibody (USP14, ab235960, 1:100 dilution, Abcam; MYC, 60003-2, 1:500 dilution, Proteintech) and fluorescently conjugated secondary antibody (goat anti-rabbit IgG, A78953, 1:2000 dilution, Invitrogen; goat anti-mouse IgG, A32727, 1:2000 dilution, Invitrogen). Nuclei were stained with DAPI. Fluorescence microscopy was used to obtain images.

### Western blot analysis

After protein extraction from the liver and cells, protein samples were separated by sodium dodecyl sulfate‒polyacrylamide gel electrophoresis and transferred to polyvinylidene difluoride membranes. The membranes were incubated overnight with the specific primary antibody and then incubated for 2 h with the appropriate horseradish peroxidase-labeled secondary antibody. Specific bands were detected by chemiluminescence assay. All antibodies used in this study are shown in Supplementary Table S2.

### RNA extraction and quantitative RT‐PCR analysis

Total RNA was extracted from the liver and cells using TRIzol (TaKaRa, Japan) and then reverse transcribed using a PrimeScript RT kit (TaKaRa, Japan). The complementary DNA obtained was subjected to quantitative RT‒PCR using SYBR Premix Ex Taq (TaKaRa, Japan). The expression levels of genes relative to β-actin mRNA levels were calculated in each sample according to the 2^−ΔΔCt^ method. All primers are shown in Supplementary Table S3.

### Immunoprecipitation assays and ubiquitination analysis

Immunoprecipitation was performed according to the Pierce™ Immunoprecipitation Kit (26149, Thermo Fisher) instructions. After HEK-293T cells were cotransfected with the corresponding plasmids, cell lysates were prepared using lysis buffer (150 mM NaCl, 10 mM HEPES, pH 7.4, 1% NP-40). The lysates were then incubated overnight at 4 °C with anti-USP14 rabbit antibody, anti-HSP90AA1 rabbit antibody, anti-CYP2E1 rabbit antibody, and protein G-conjugated agarose, or Flag and Myc affinity agarose. Beads containing bound protein were washed six times using IP Wash Buffer and eluted with glycine. The eluate was mixed with 5× loading buffer and boiled at 95 °C for 10 min for immunoblotting. For ubiquitination analysis, the indicated plasmids were cotransfected into 293 T cells, which were then lysed in precooled IP lysis buffer containing SDS. The subsequent steps were identical to those for co-IP.

### Plasmid constructs

The cDNAs of mouse USP14, HSP90AA1, and CYP2E1 were amplified using PCR and cloned into the pEnCMV-MCS-3 × FLAG-SV40-Neo vector or pCMV-MCS-3 × Myc-Neo vector to obtain overexpression vectors with different tags. An active site mutant of USP14 (USP14 C114A) and a truncation mutant of USP14 lacking the UBL were constructed with reference to previously described methods [24]. Primer sequences for plasmid constructs are listed in Table S1. Ubiquitin (Ub) expression vectors with HA tags were obtained from the Miaoling Plasmid Platform (Wuhan, China).

### RNA sequencing and data processing

Total RNA was extracted from each group of livers using TRIzol reagent (Ambion/Invitrogen, USA), followed by stringent quality control of the RNA samples and construction of cDNA libraries. After passing library inspection, the library was pooled and sequenced by illumina NovaSeq 6000 (illumina, USA) according to the effective concentration and the target downstream data volume required. Gene expression values (FPKM) of all samples were analyzed by PCA. Hierarchical clustering was displayed using the weighted two-group arithmetic method (UPGMA) and the HLLUST function of the R package. KEGG pathway enrichment analysis of all differential genes was performed by Fisher’s exact test. pathways with *P* < 0.05 were defined as significantly enriched pathways.

Gene set enrichment analysis used KEGG as a predefined gene set to compare HFD-pAd-CMV and HFD-pAd-USP14 based on their expression levels. Enrichment scores (ES) were then calculated to estimate the significance level (*P* value) of the ES. The ES of each gene set was standardized to obtain a normalized enrichment score (NES). The false positive rate was controlled by calculating the false discovery rate (FDR) value; gene sets with an FDR < 0.25 were considered statistically significant.

### Statistical analysis

Statistical analyses were performed using GraphPad Prism 8.0 or SPSS 21.0 (IBM Corp., Armonk). After the data passed the normal distribution test, Student’s *t* test was used to analyze significant differences between the two groups, and by one-way analysis of variance (ANOVA) followed by Tukey–Kramer test or Dunnett’s test for more than two groups. Data are shown as the mean ± standard deviation (SD). *p* values < 0.05 were considered statistically significant. No statistical methods were used to predetermine the sample size. No blinding was used during experiments and outcome analysis.

## Supplementary information

Supplementary Material

Original Data File

Reproducibility checklist

## Data Availability

All study data are included in the article.
