# Supplementary material for: USP14 governs CYP2E1 to promote nonalcoholic fatty liver disease through deubiquitination and stabilization of HSP90AA1
Source: Cell Death Dis. 2023 Aug 26;14(8):566. doi: 10.1038/s41419-023-06091-6 (PMC10460448; doi:10.1038/s41419-023-06091-6)
Supplement: Supplementary file 1 — Supplementary Material [file 41419_2023_6091_MOESM1_ESM.docx]

**Supplementary Figure and Figure Legends**


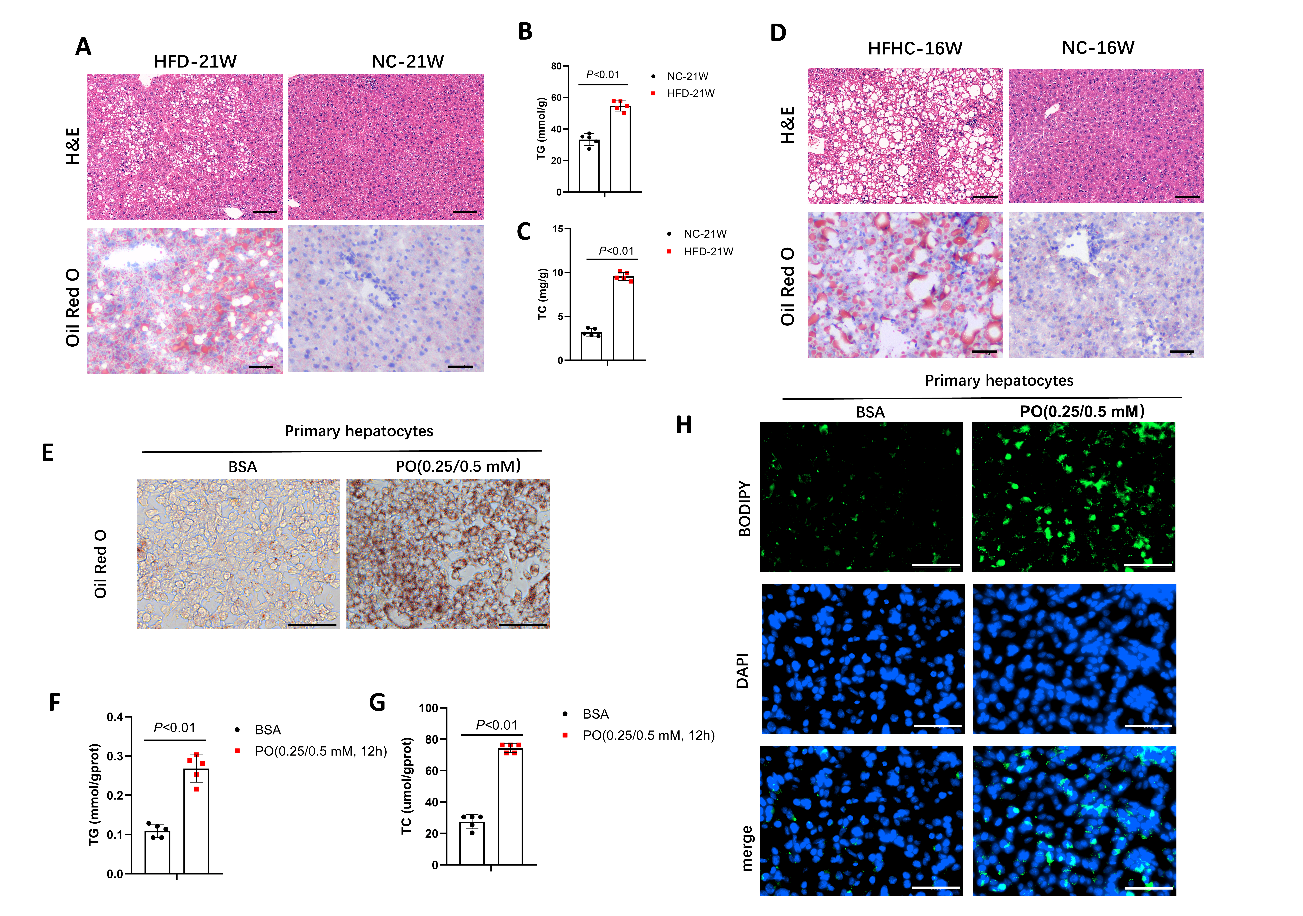


**Supplementary** **Fig. S1: Effects of HFD, HFHC and PO treatments on mouse liver or primary hepatocytes.** (A) H&E and Oil Red O staining of livers from HFD or normal diet mice. Scale bar, 50 μm. (B, C) Analysis of hepatic TG and TC levels in HFD or normal diet mice (n=5). (D) Liver H&E and Oil Red O staining in HFHC or normal diet mice. Scale bar, 50 μm. (E) Oil-red O staining of mouse primary hepatocytes after PO treatment for 12 hours. Scale bar, 100 μm. (F, G) TG and TC levels in primary mouse hepatocytes 12 h after PO treatment (n=5). (H) BODIPY staining of primary mouse hepatocytes 12 h after PO treatment. Scale bar, 100 μm.


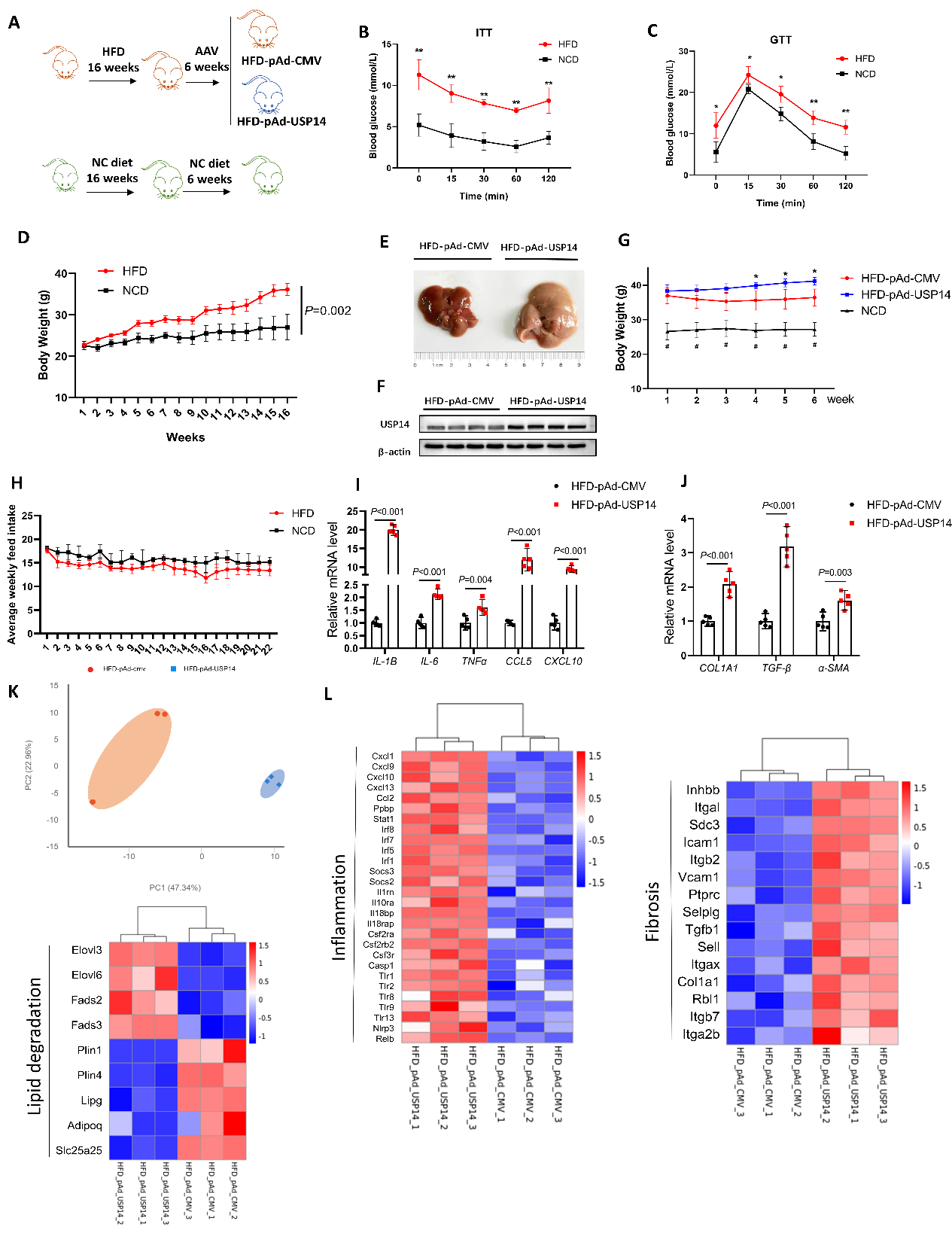


**Supplementary Fig. S2: Effect of mouse liver USP14 overexpression on the course of NAFLD.** (A) Schematic representation of mice grouped and treated. (B, C) GTT and ITT results in mice after 16 weeks of HFD or normal diet treatment (n=30). * *P*<0.05 for the HFD group compared to the NCD group; ** *P*<0.01 for the HFD group compared to the NCD group. (D) Body weights of mice treated with HFD or normal diet for 16 weeks. (E) Representative liver photographs of mice after pAd-CMV and pAd-USP14 adenovirus injection under HFD treatment. (F) Liver USP14 protein levels in mice after pAd-CMV and pAd-USP14 adenovirus injection under HFD treatment (n=4). (G) Trends in body weight change over 6 weeks of adenovirus injection (n=5). * *P*<0.05 for the HFD-pAd-CMV compared to the HFD-pAd-USP14 group. # *P*<0.01 for the NCD compared to the HFD-pAd-CMV group. (H) Average weekly feed intake of all mice. (I, J) Expression of liver inflammation-related genes and fibrosis-related genes in mice after pAd-CMV and pAd-USP14 adenovirus injections under HFD treatment (n=5). (K) Principal component analysis showed the distribution of HFD diet-induced liver samples from each group according to gene expression profiles. (L) Heat map showing the distribution of activation of lipid metabolism, inflammation and fibrosis-related genes in the liver.


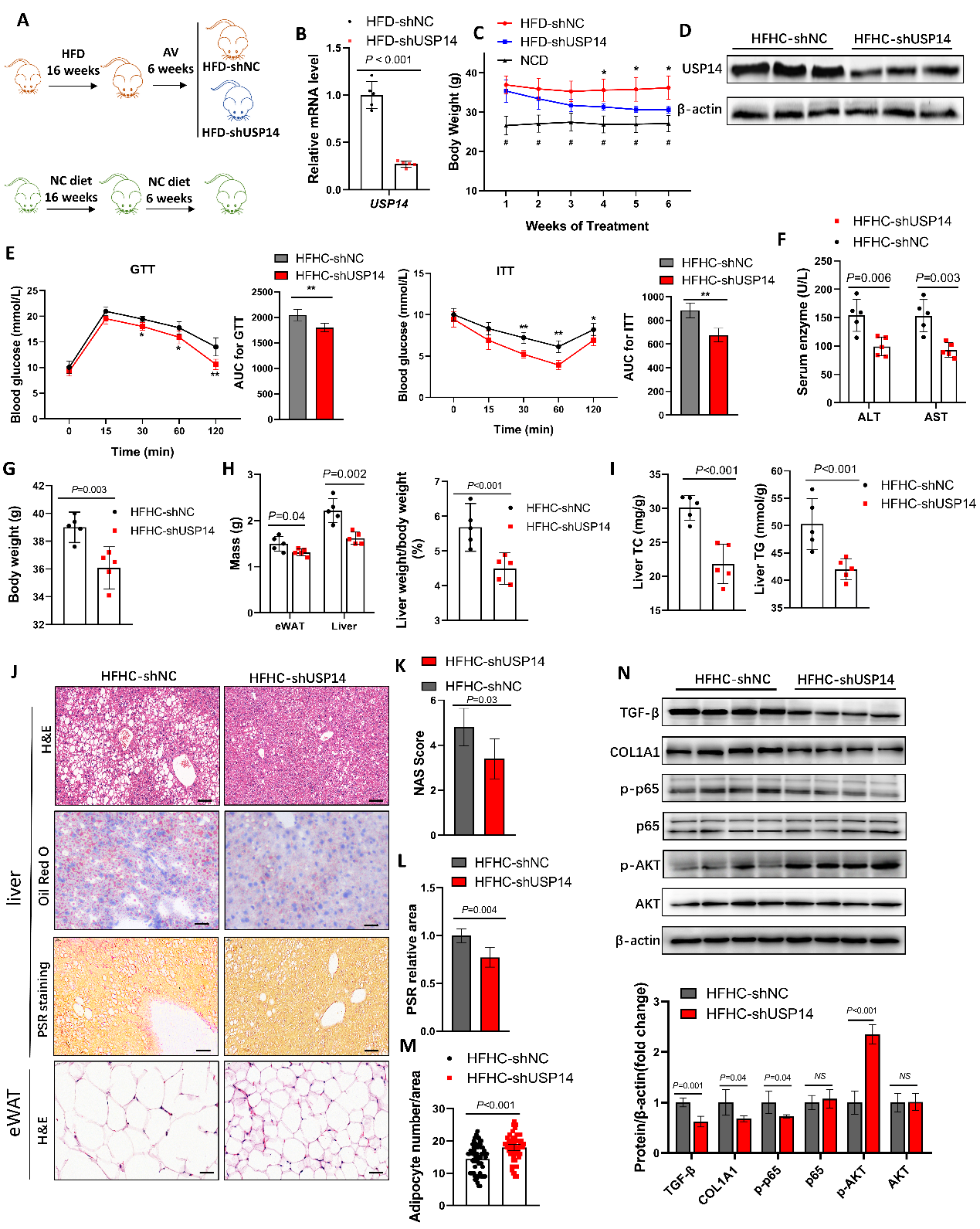


**Supplementary Fig. S3: Hepatic USP14 knockdown alleviates HFHC-induced NASH.** (A) Schematic representation of mice grouped and treated. (B) USP14 mRNA levels in livers of shNC or shUSP14 adenovirus-treated mice. (C) Trends in body weight change over 6 weeks of adenovirus injection (n=5). * *P*<0.05 for the HFD-shNC compared to the HFD-shUSP14 group. # *P*<0.01 for the NCD compared to the HFD-shNC group. (D) Liver USP14 protein levels in mice. (E) GTT and ITT analysis of mice five weeks after shNC or shUSP14 adenovirus injection (n=12). AUC, area under the curve. * *P*<0.05 for the HFHC-shNC compared to the HFHC-shUSP14 group; ** *P*<0.01 for the HFHC-shNC compared to the HFHC- shUSP14 group. (F, G) Effect of shNC or shUSP14 adenovirus injection on serum AST and ALT levels and body weight in mice. (H) Tissue weight and LW/BW ratio of mice in each group. (I) Liver TG and TC levels of mice in each group. (J) H&E staining, oil red O staining and PSR staining from the livers or eWAT. (K, L, M) Liver NAS score, liver relative PSR area and eWAT adipocyte counts from each group of mice. (M) Expression of inflammation-related proteins, fibrosis-related proteins and insulin sensitivity-related proteins in the liver of mice in each group. *NS*, not signiﬁcant.


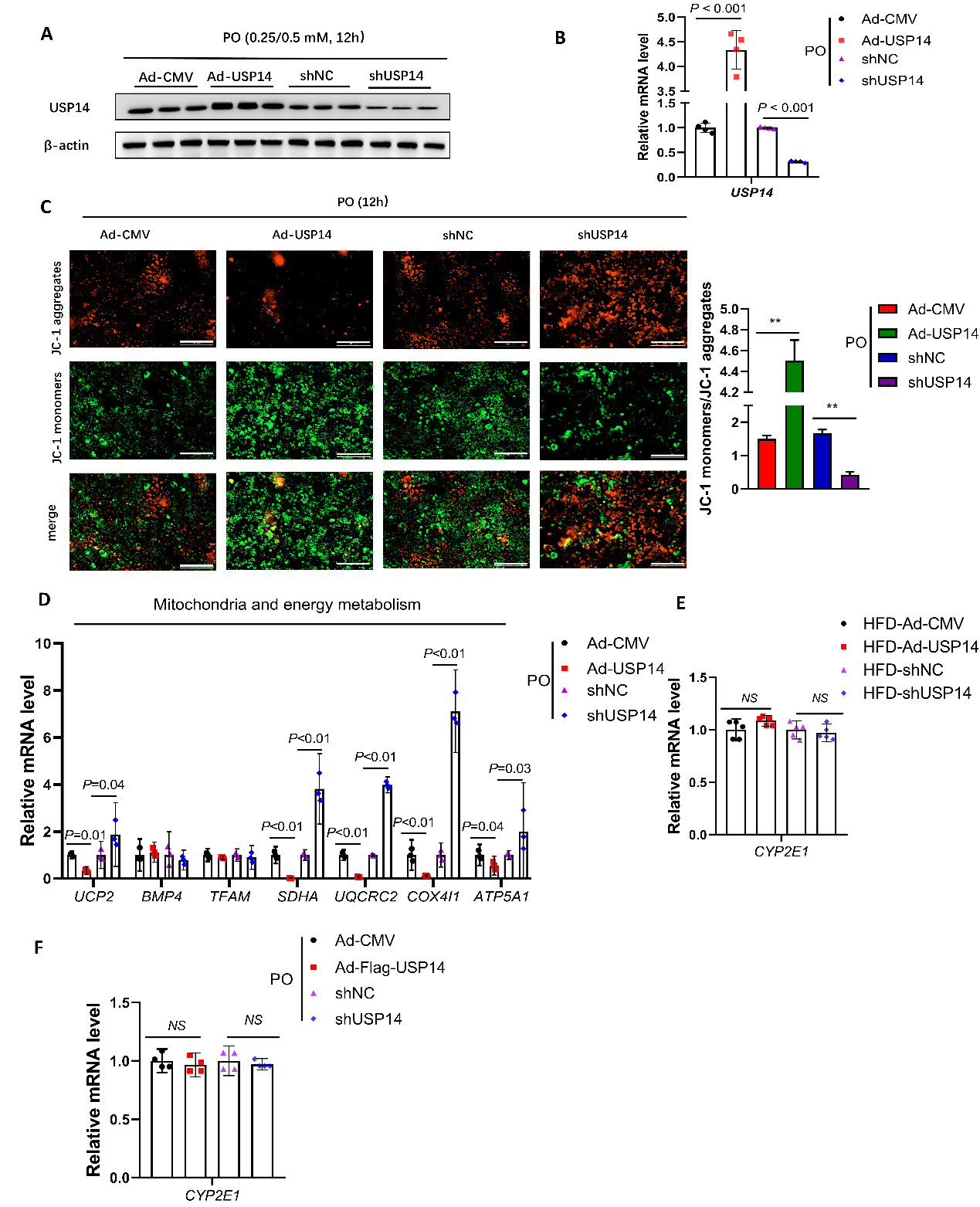


**Supplementary Fig. S4: Effect of USP14 overexpression or knockdown in mouse hepatocytes.** (A, B) Effect of AML12 cells transfected with adenovirus-associated vector under PO treatment on USP14 protein (n=3) and mRNA levels (n=4). (C) Mitochondrial membrane potential assay of AML12 cells after transfection with adenovirus-associated vector under PO treatment. Scale bar, 200 μm. (D) Detection of mitochondrial and energy metabolism-related gene expression in AML12 cells transfected with the corresponding vector of adenovirus under PO treatment. (E) mRNA levels of CYP2E1 from the liver of each group of mice. (F) Effect of AML12 cells transfected with adenovirus-associated vector under PO treatment on mRNA levels of CYP2E1 (n=4). *NS* represents a non-significant difference between the two groups. ** represents highly significant difference between the two groups.


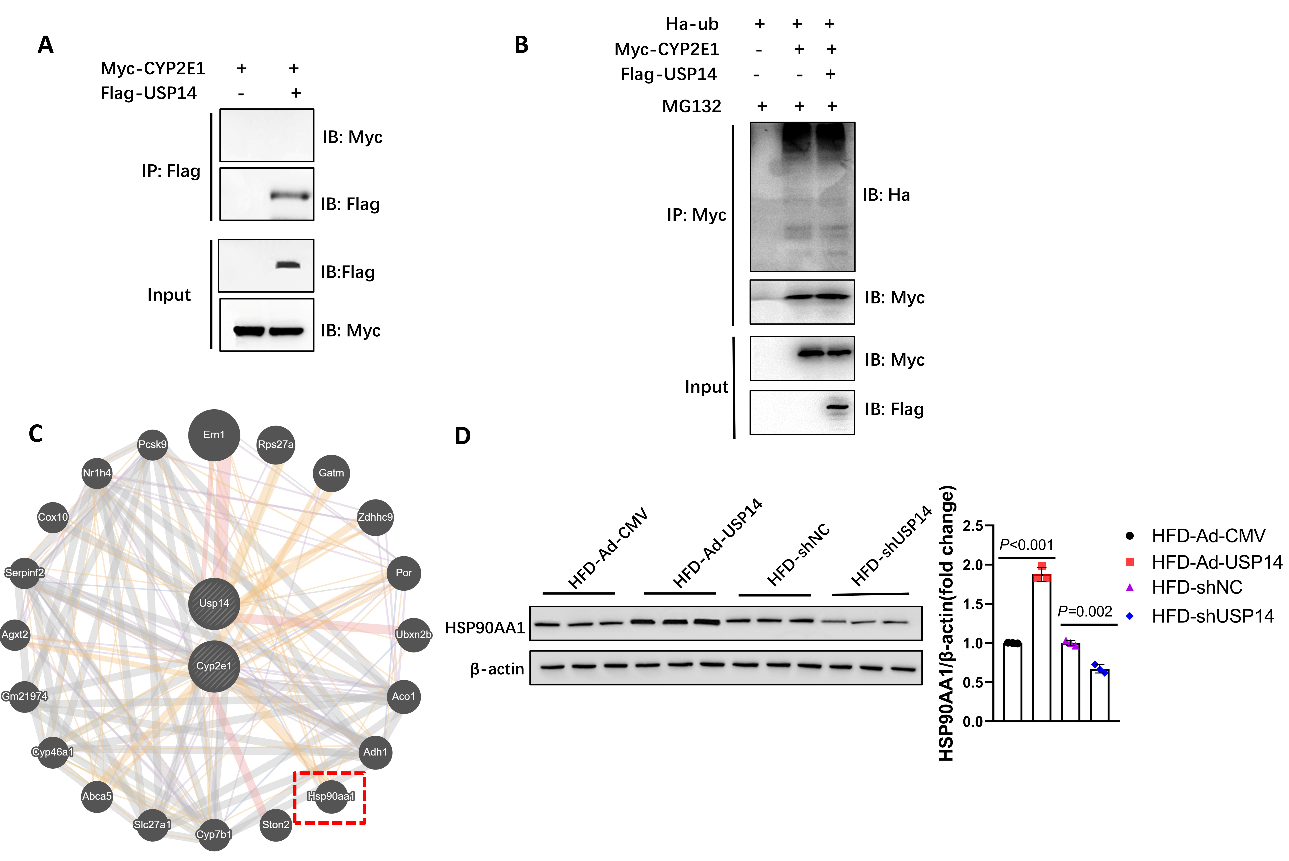


**Supplementary Fig. S5: Analysis of the interactions between USP14 and CYP2E1.** (A, B) Co-IP analysis of the interaction and ubiquitination between USP14 and CYP2E1 after HEK-293T cells were transfected with the corresponding plasmids. (C) GeneMANIA website to predict potential target proteins for USP14 and CYP2E1 interaction. (D) HAP90AA1 protein levels in liver after USP14 overexpression and knockdown.


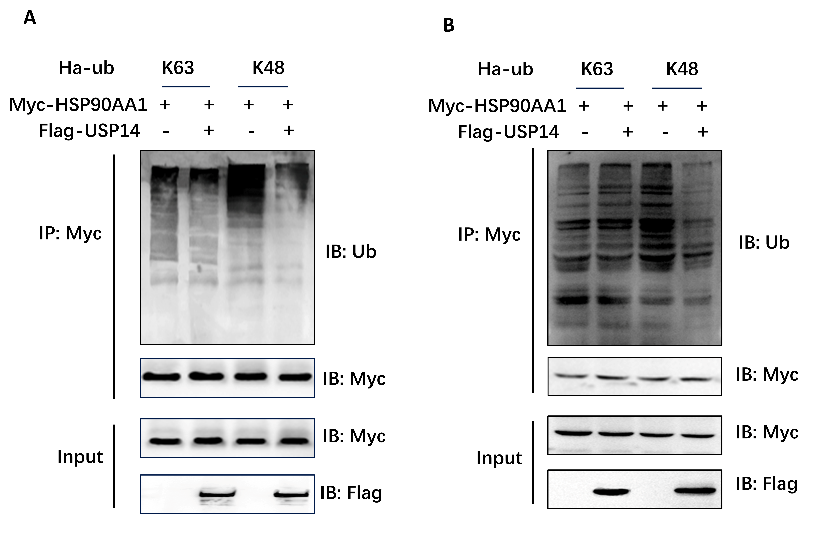


**Supplementary Fig. S6: Analysis of deubiquitination between USP14 and HSP90AA1.** (A, B) Identification of the type of HAP90AA1 deubiquitination by USP14. Two replicates of the results.


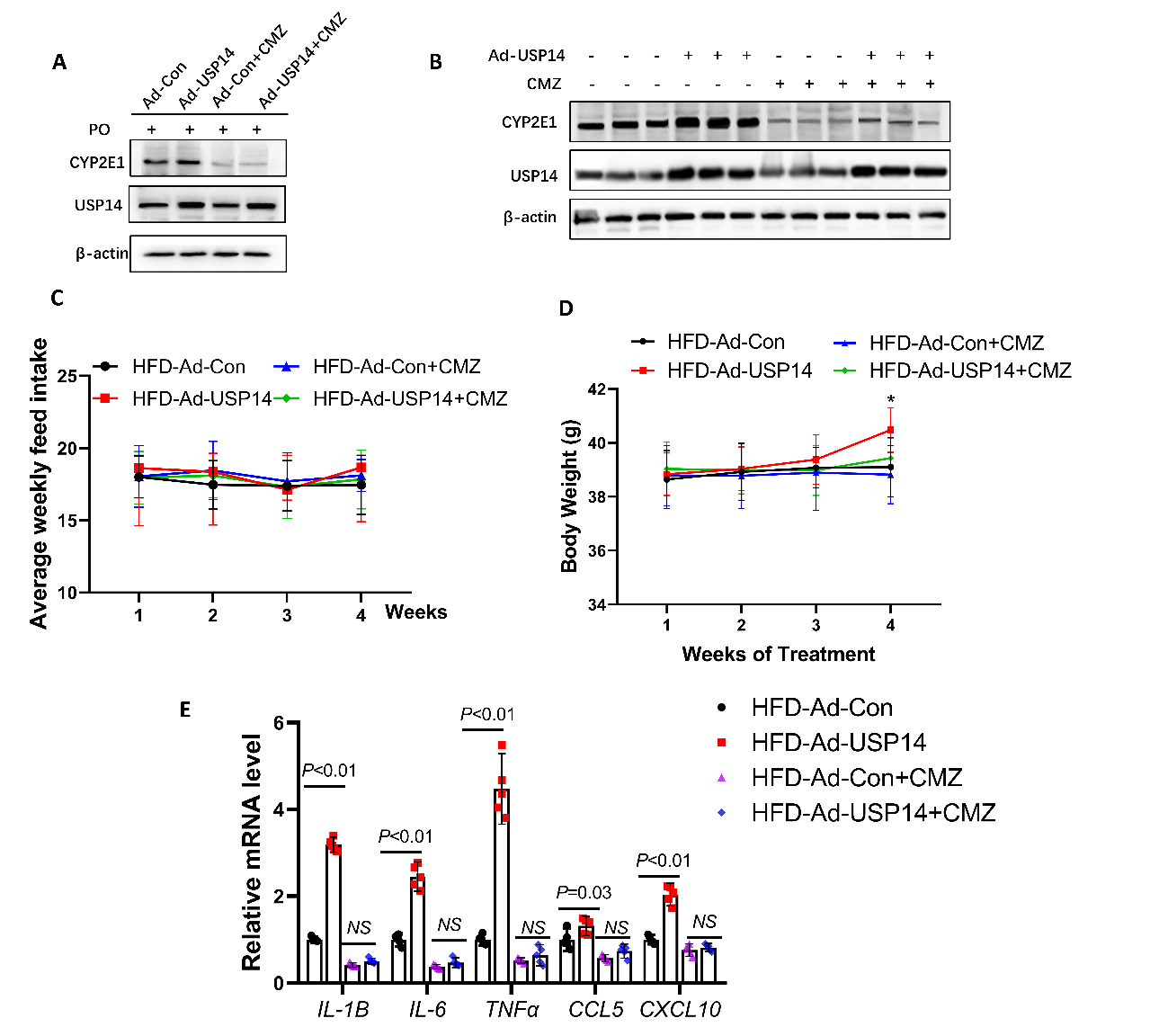


**Supplementary Fig. S7: The function of USP14 in LPO and inflammation depends on CYP2E1.** (A) Protein expression of USP14 and CYP2E1 in AML12 cells under CMZ treatment and corresponding plasmid transfection. (B) Effect of CMZ and Ad-USP14 adenovirus injection in mice on hepatic CYP2E1 and USP14 protein expression. (C, D) Feed intake and body weight of each group (n=5). * *P*<0.05 for the HFD-Ad-Con compared to the HFD-Ad-USP14 group. There was no difference between the other groups. (E) Effect of CMZ and Ad-USP14 adenovirus injections in mice on liver inflammatory gene expression (n=5).

**Supplementary Table S1** Primers used to generate constructs in this study

| **Gene name** | **Primer** |
| --- | --- |
| pAd-USP14 | F: CCGGAATTCATGCCACTCTACTCTGTTACAGTAA |
|  | R: CGGGGTACCCTGTTCACTTTCTTCTTCCATTATT |
| Flag-USP14 | F: AAGCTTGGTACCGAGCTCGGATCCGCCACCATGCCACTCTACTCTGTTAC |
|  | R: CTGTGCTGGATATCTGCAGAATTCCTGTTCACTTTCTTCTTCC |
| Flag-USP14 C114A | F1: TTGGTACCGAGCTCGAATTCGCCACCATGCCACTATACTCTGTTAC |
|  | R1: GTAGCATTCATGTAAGCAGTGTTACCAAGGTT |
|  | F2: AACCTTGGTAACACTGCTTACATGAATGCTAC |
|  | R2: TGGTCTTTGTAGTCGGATCCCTGTTCACTTTCTTCTTCCATT |
| Flag-USP14 ∆UBL | F: TTGGTACCGAGCTCGAATTCGCCACCATGGAACCCTCTGCTAAAACTGTCTTCGTAG |
|  | R: TGGTCTTTGTAGTCGGATCCCTGTTCACTTTCTTCTTCCATT |
| MYC-HSP90AA1 | F: AAGCTTGGTACCGAGCTCGGATCCGCCACCATGCCTGAGGAAACCCAG |
|  | R: CTGTGCTGGATATCTGCAGAATTCGTCTACTTCTTCCATGCGTG |
| MYC-CYP2E1 | F: AGTCCAGTGTGGTGGAATTCGCCACCATGGCGGTTCTTGGCATCACCGTTG |
|  | R: ATTAGTTTTTGTTCCTCGAGTGAACGAGGAATGACACAGAGTTTAAATTCG |
| **Target sequences** | **Sequence (5'-3')** |
| shUSP14#1  (mouse) | CCTGCTTACTTAACTATTCAA |
| shUSP14#2  (mouse) | GAGAAGTTTGAAGGTGTAGAA |
| HSP90AA1 siRNA  (mouse) | CTAGGTATTGATGAGGATGAT |
|  |  |

**Supplementary Table S2** Information on all primary antibodies used in this study

| **ANTIBODIES** | **SOURCE** | **IDENTIFIER** |
| --- | --- | --- |
| USP14 | CY6893 | ABWAYS |
| CYP2E1 | CY6754 | ABWAYS |
| HSP90AA1 | ABS115111 | ABSIN |
| FASN | CY6597 | ABWAYS |
| PPARG | AB209350 | ABCAM |
| ATGL | 2138 | CST |
| P65 | 8242 | CST |
| P-P65 | 3033 | CST |
| TGF-β | YEASEN | 30013ES50 |
| COL1A1 | ABWAYS | CY5120 |
| AKT | AB8805 | ABCAM |
| P-AKT | 4060 | CST |
| β-ACTIN | AC026 | ABCLONAL |
| FLAG | 66008-4 | PROTEINTECH |
| MYC | 66006-2 | PROTEINTECH |
| HA | 60003-2 | PROTEINTECH |
| Ub | AB134953 | ABCAM |

**Supplementary Table S3** The RT-PCR primers used in this study

| **Gene name** | **Primer** |
| --- | --- |
| *m-USP14* | F: ATGCACTCTCTGTAGT; |
|  | R: AACACATTGGTTCAG |
| *m-CYP2E1* | F: GGACCTTTCCCAATTCCTTTCTT; |
|  | R: TCTTGTGGTTCAGTAGCACCT |
| *m-IL-1B* | F: GCAACTGTTCCTGAACTCAACT; |
|  | R: ATCTTTTGGGGTCCGTCAACT |
| *m-IL6* | F: TAGTCCTTCCTACCCCAATTTCC; |
|  | R: TTGGTCCTTAGCCACTCCTTC |
| *m-CCL5* | F: GCTGCTTTGCCTACCTCTCC; |
|  | R: TCGAGTGACAAACACGACTGC |
| *m-CXCL10* | F: CCAAGTGCTGCCGTCATTTTC; |
|  | R: GGCTCGCAGGGATGATTTCAA |
| *m-TNFα* | F: CCCTCACACTCAGATCATCTTCT; |
|  | R: GCTACGACGTGGGCTACAG |
| *m-COL1A1* | F: TGCTAACGTGGTTCGTGACCGT |
|  | R: ACATCTTGAGGTCGCGGCATGT |
| *m-TGF-β* | F: ATTTGGAGCCTGGACACACA |
|  | R: GAGCGCACAATCATGTTGGA |
| *m-α-SMA* | F: CCCAGACATCAGGGAGTAATGG |
|  | R: GAGCGCACAATCATGTTGGA |
| *m-UCP2* | F: ATGGTTGGTTTCAAGGCCACA |
|  | R: CGGTATCCAGAGGGAAAGTGAT |
| *m-BMP4* | F: TTCCTGGTAACCGAATGCTGA |
|  | R: CCTGAATCTCGGCGACTTTTT |
| *m-TFAM* | F: ATTCCGAAGTGTTTTTCCAGCA |
|  | R: TCTGAAAGTTTTGCATCTGGGT |
| *m-SDHA* | F: GGAACACTCCAAAAACAGACCT |
|  | R: CCACCACTGGGTATTGAGTAGAA |
| *m-UQCRC2* | F: AAAGTTGCCCCGAAGGTTAAA |
|  | R: GAGCATAGTTTTCCAGAGAAGCA |
| *m-COX4I1* | F: ATTGGCAAGAGAGCCATTTCTAC |
|  | R: CACGCCGATCAGCGTAAGT |
| *m-ATP5A1* | F: TCTCCATGCCTCTAACACTCG |
|  | R: CCAGGTCAACAGACGTGTCAG |
| *m-beta-actin* | F: GGCTGTATTCCCCTCCATCG; |
|  | R: CCAGTTGGTAACAATGCCATGT |
