# Supplementary material for: USP14 governs CYP2E1 to promote nonalcoholic fatty liver disease through deubiquitination and stabilization of HSP90AA1
Source: Cell Death Dis. 2023 Aug 26;14(8):566. doi: 10.1038/s41419-023-06091-6 (PMC10460448; doi:10.1038/s41419-023-06091-6)

USP14 governs CYP2E1 to promote nonalcoholic fatty liver disease through deubiquitination and stabilization of HSP90AA1

Dongqin Wei, Xin Tian, Longbo Zhu, Han Wang, Chao Sun

Fig. 1B

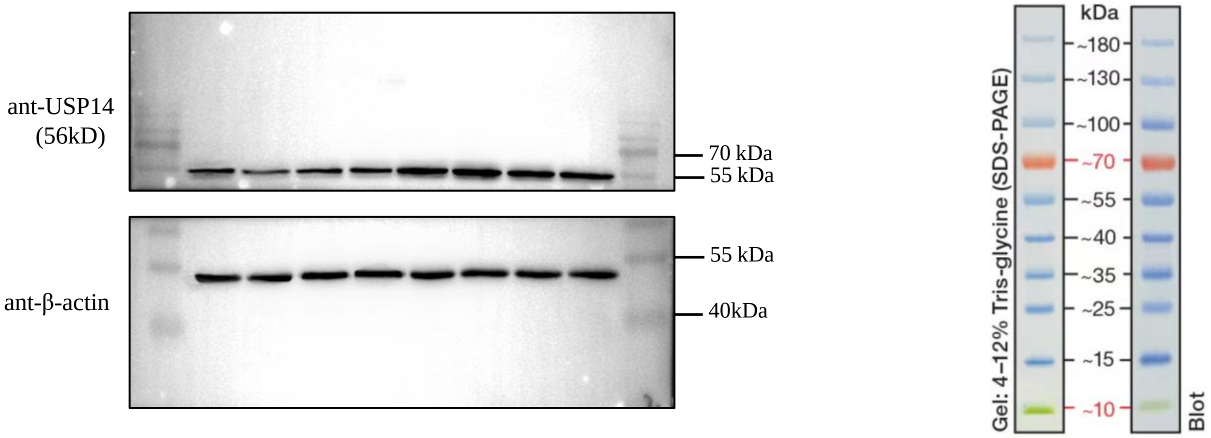

Fig. 1D

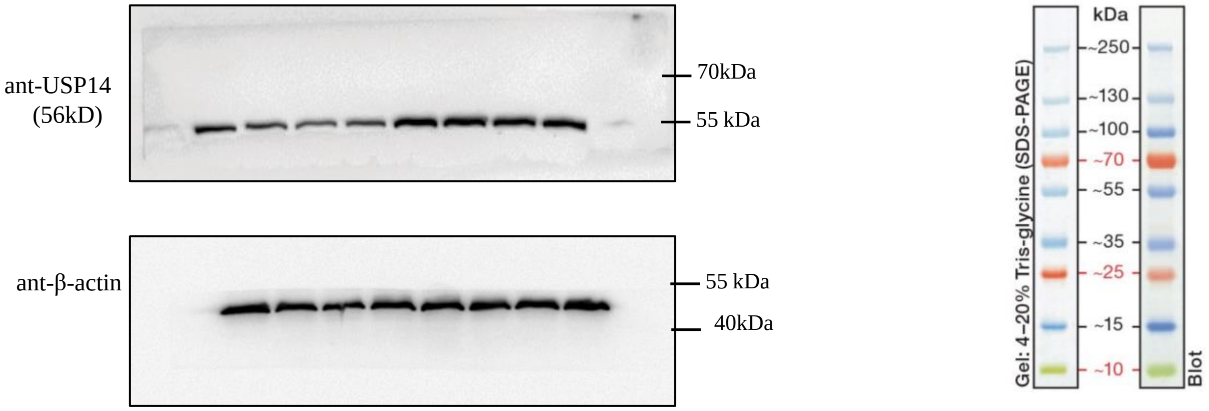

Fig. 1F

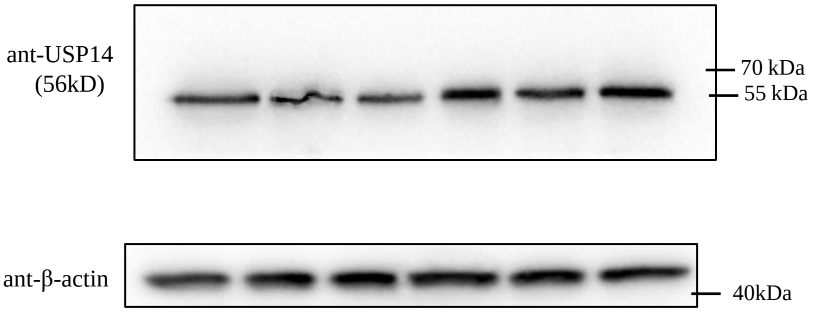

Fig. S2F

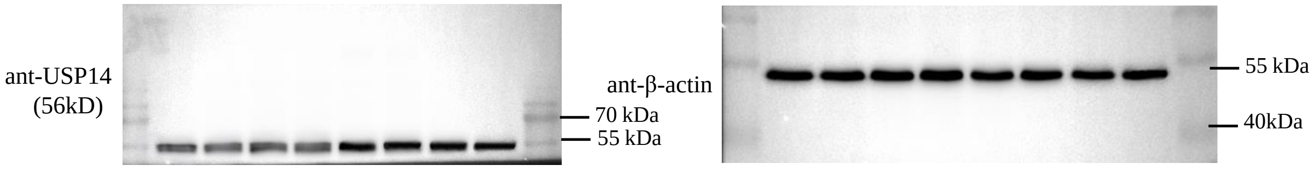

Fig. 2J

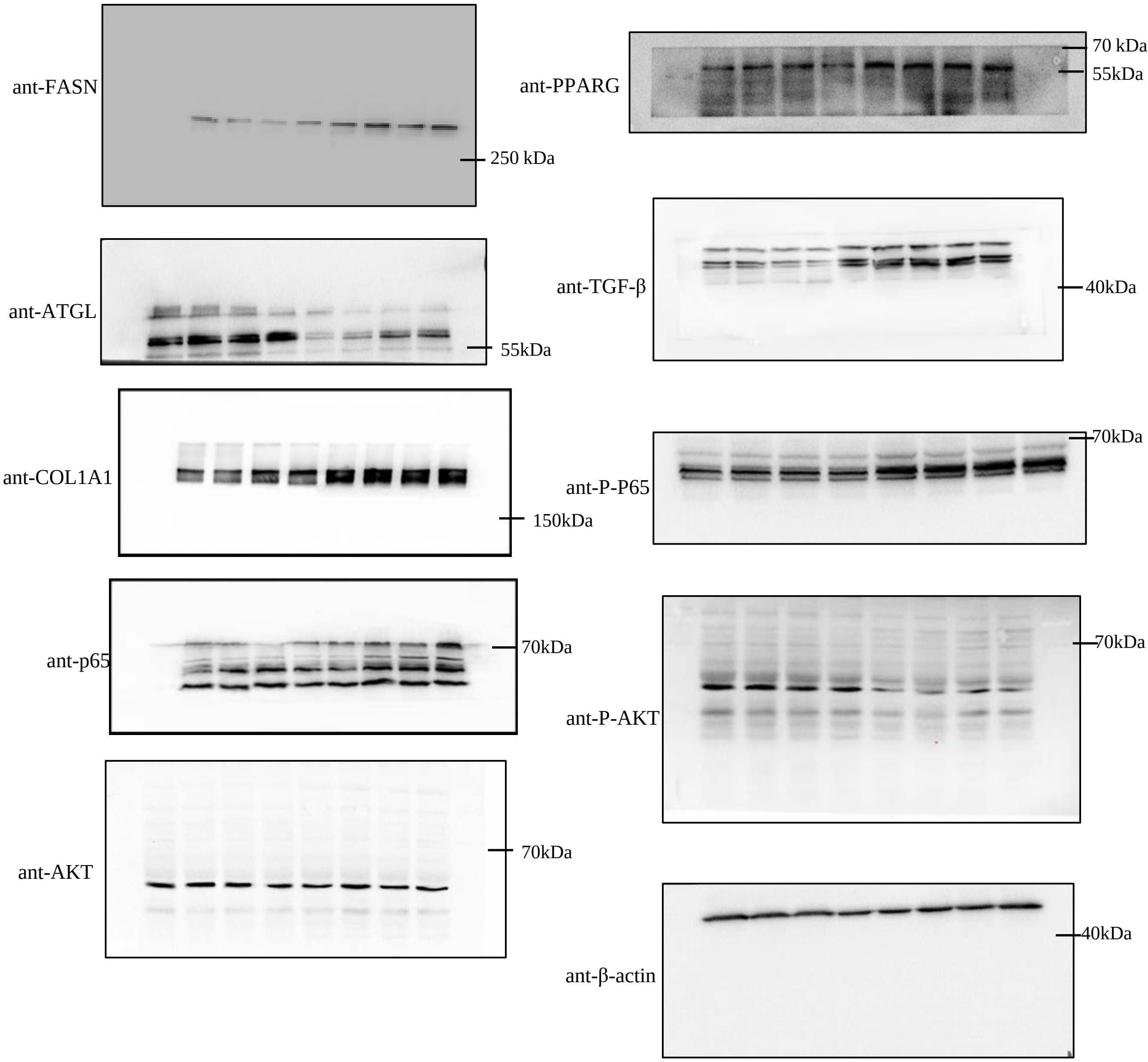

Fig. 3A

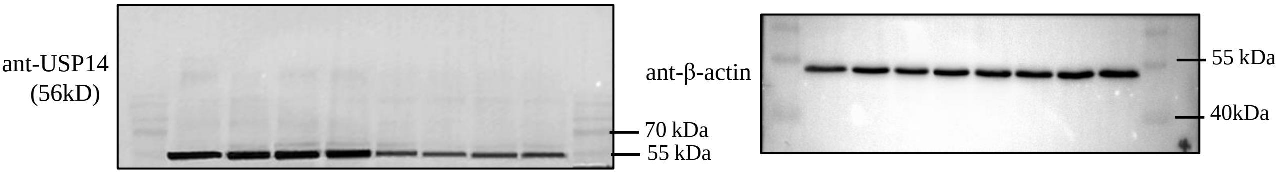
ant-USP14
(56kD)
70 kDa
55 kDa
ant-β-actin
55 kDa
40kDa

Fig. 3M

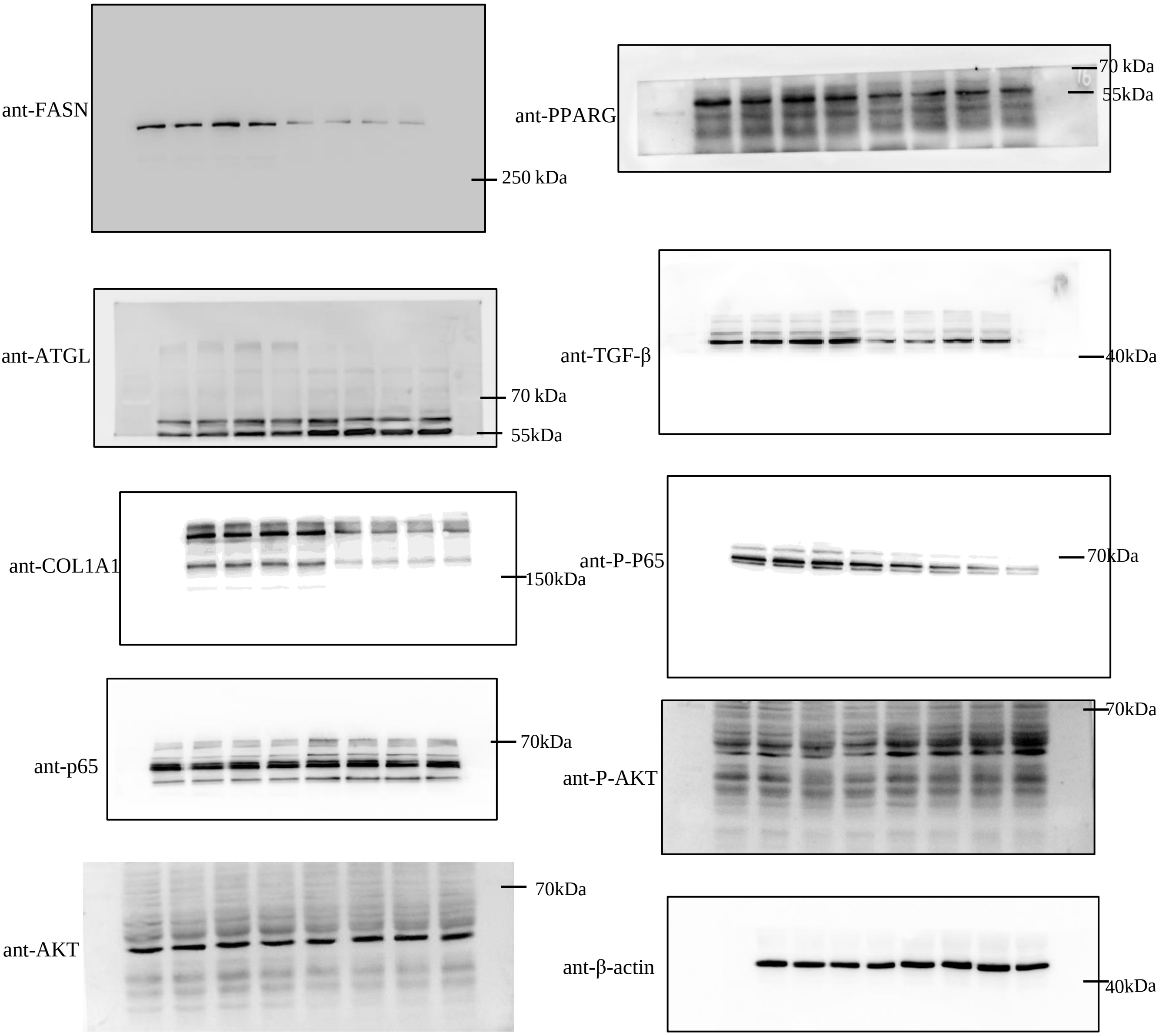
ant-FASN
250 kDa
ant-PPARG
70 kDa
55kDa
ant-ATGL
70 kDa
55kDa
ant-TGF-β
40kDa
ant-COL1A1
150kDa
ant-P-P65
70kDa
ant-p65
70kDa
ant-P-AKT
70kDa
ant-AKT
70kDa
ant-β-actin
40kDa

Fig. S3N

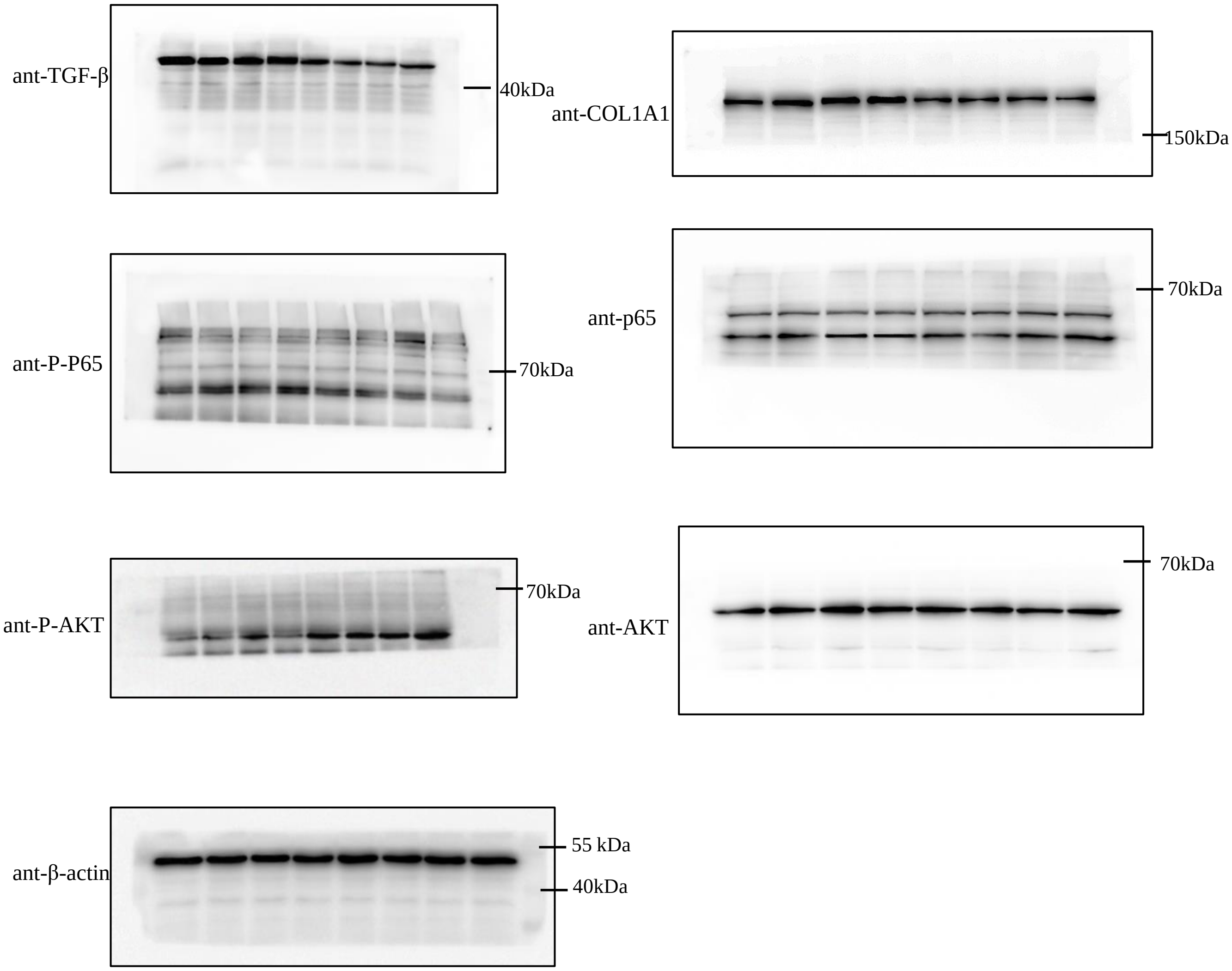
ant-TGF-β
40kDa
ant-COL1A1
150kDa
ant-P-P65
70kDa
ant-p65
70kDa
ant-P-AKT
70kDa
ant-AKT
70kDa
ant-β-actin
55 kDa
40kDa

Fig. S4A

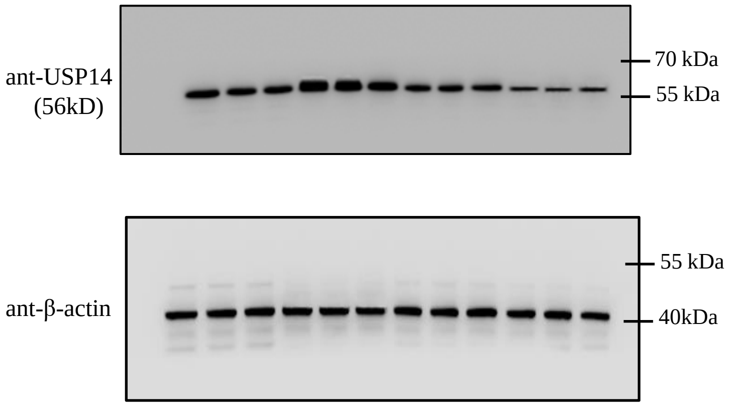

Fig. 4J

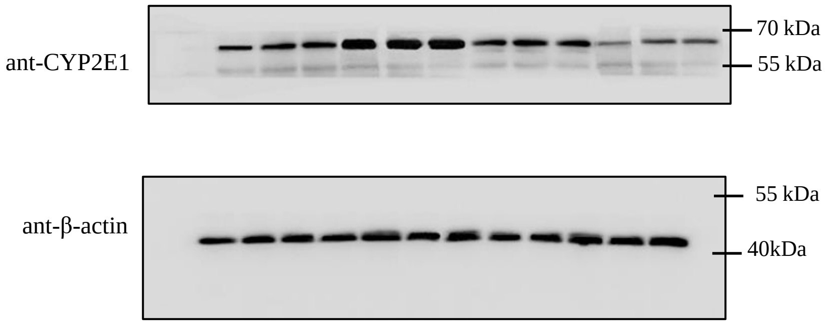

Fig. 4K

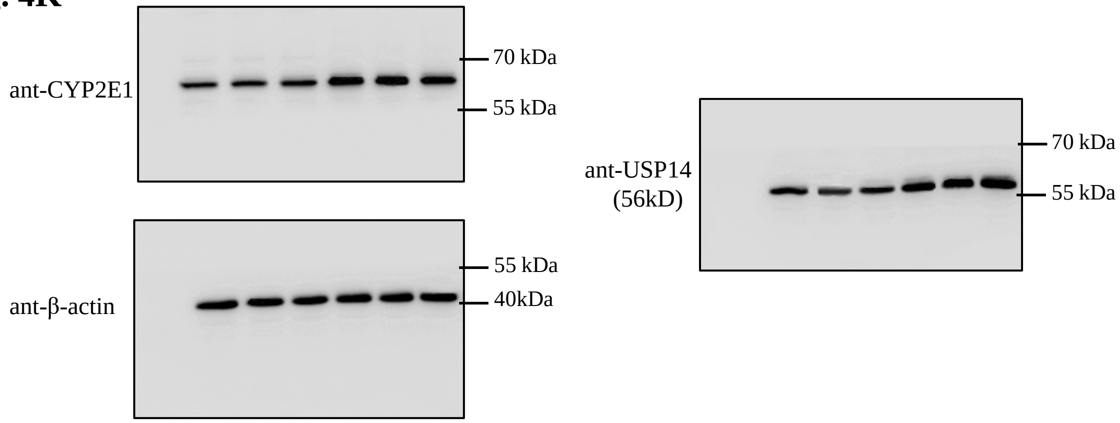

Fig. 4L

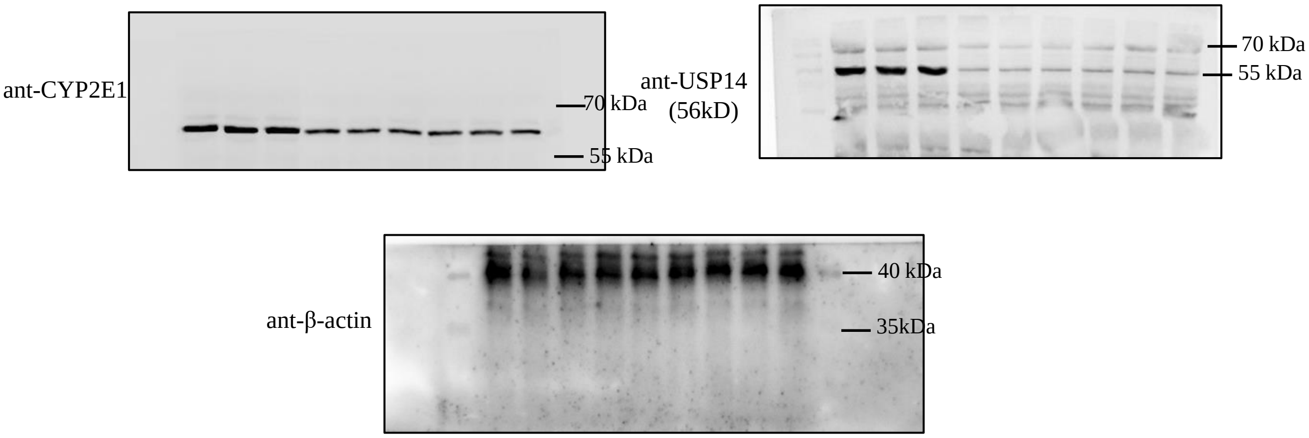

Fig. 4M

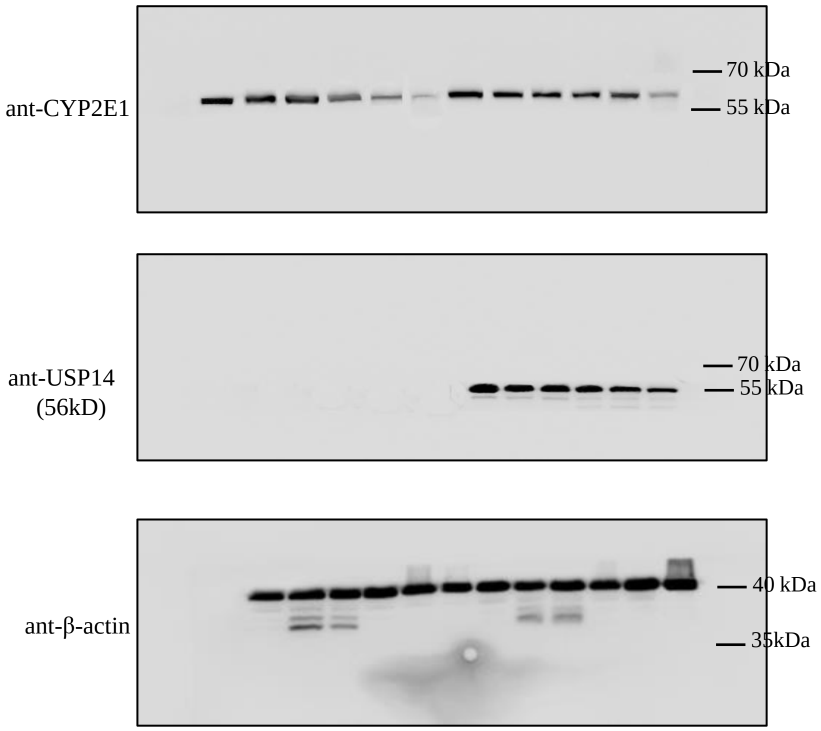

Fig. S5A

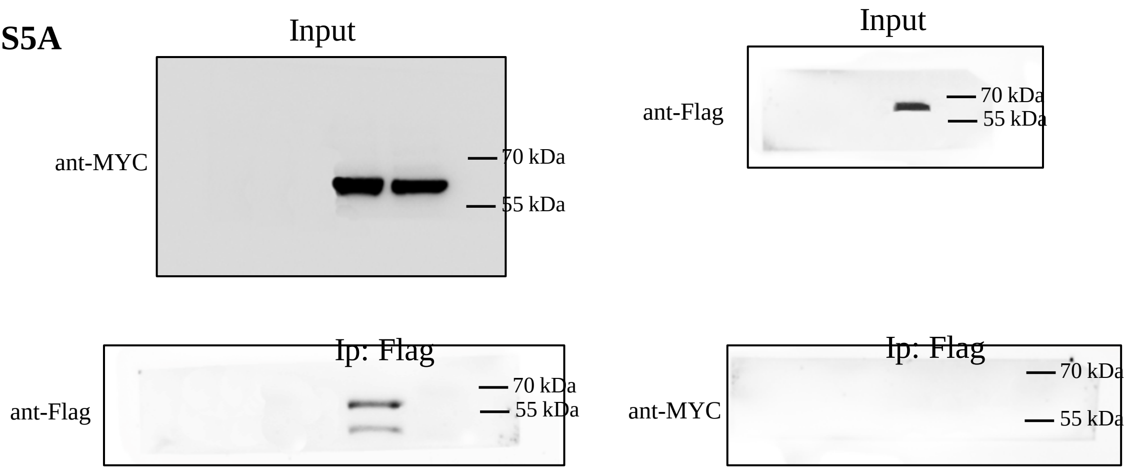

Fig. 5A

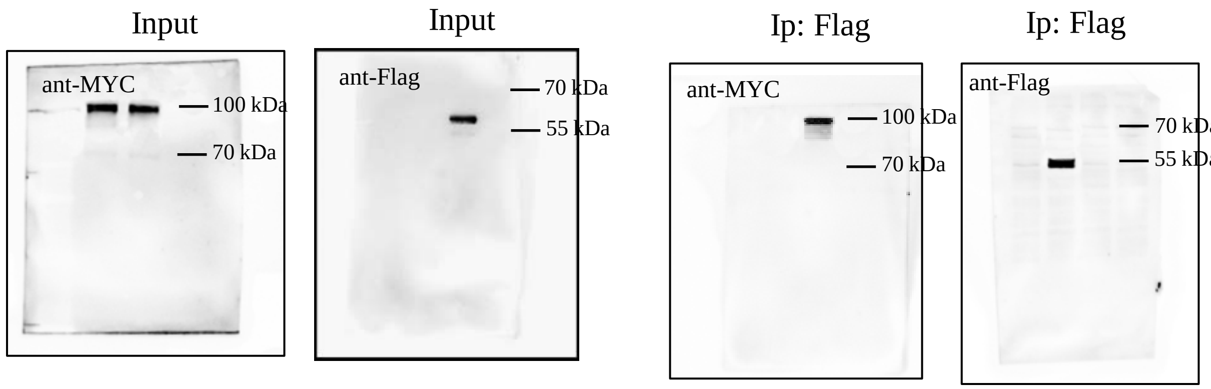

Fig. 5B

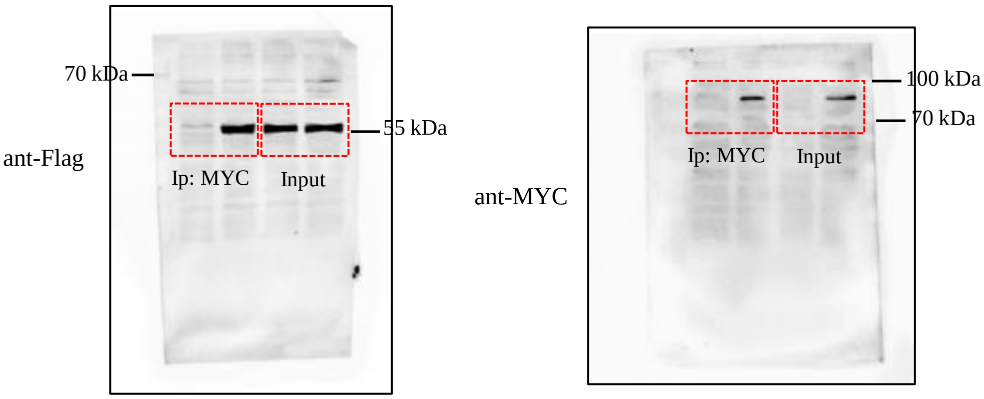

Fig. 5C

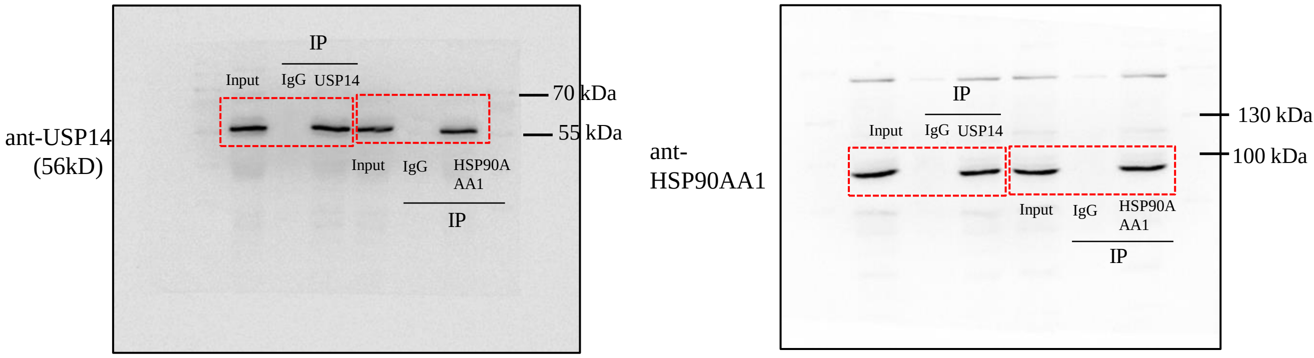

Fig. 5E

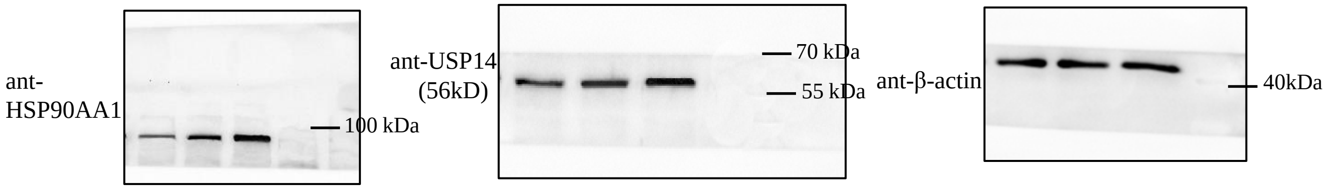

Fig. 5F

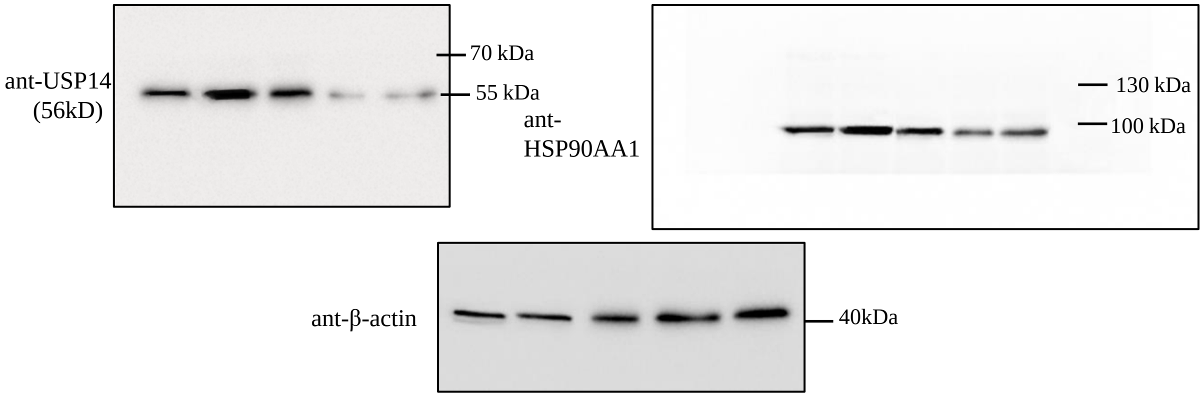

Fig. S5B

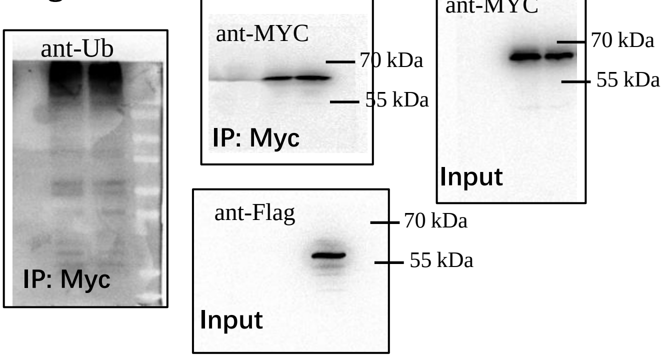

Fig. S5D

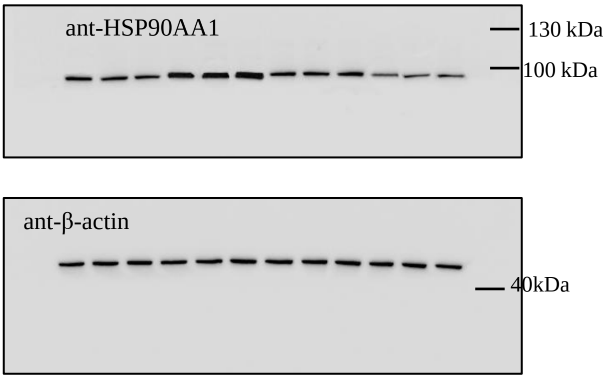

Fig. 5G

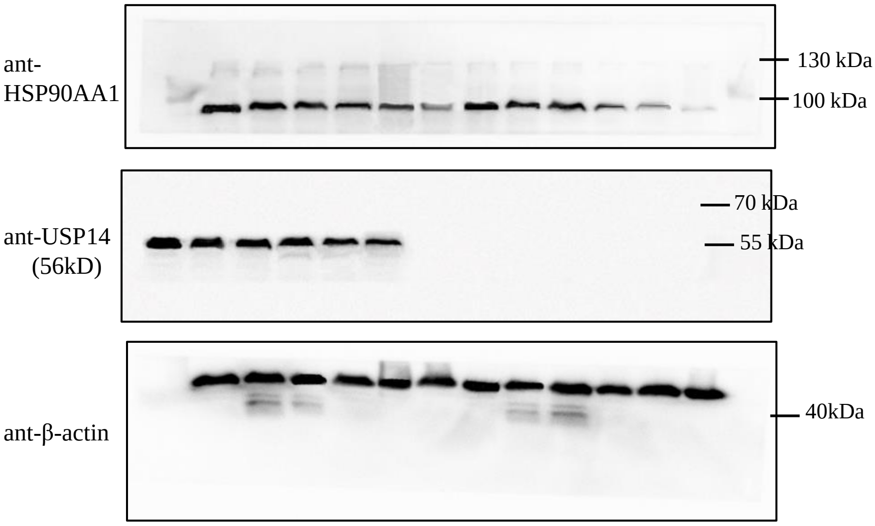

Fig. 5H

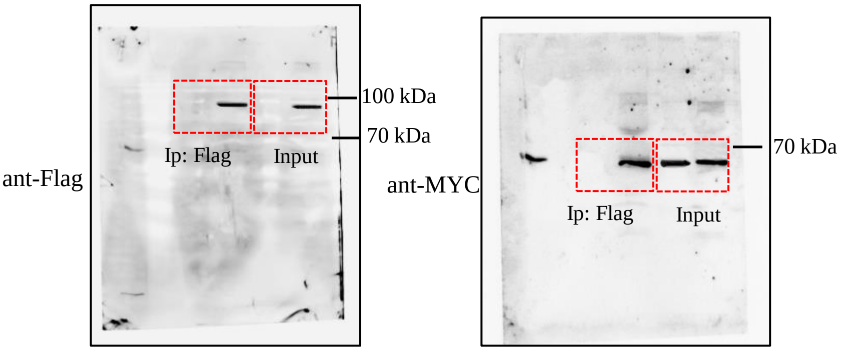

Fig. 5I

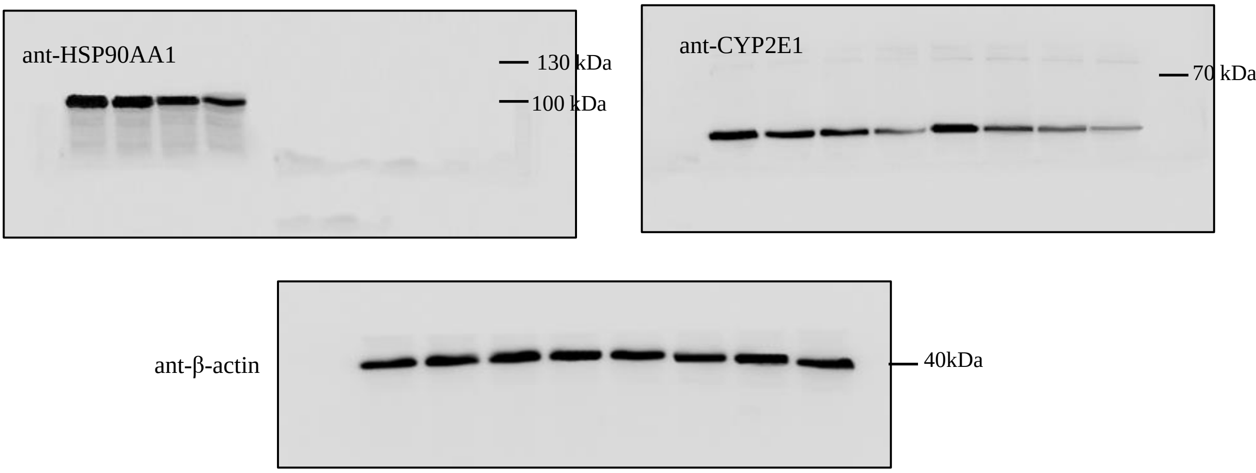

Fig. 5J

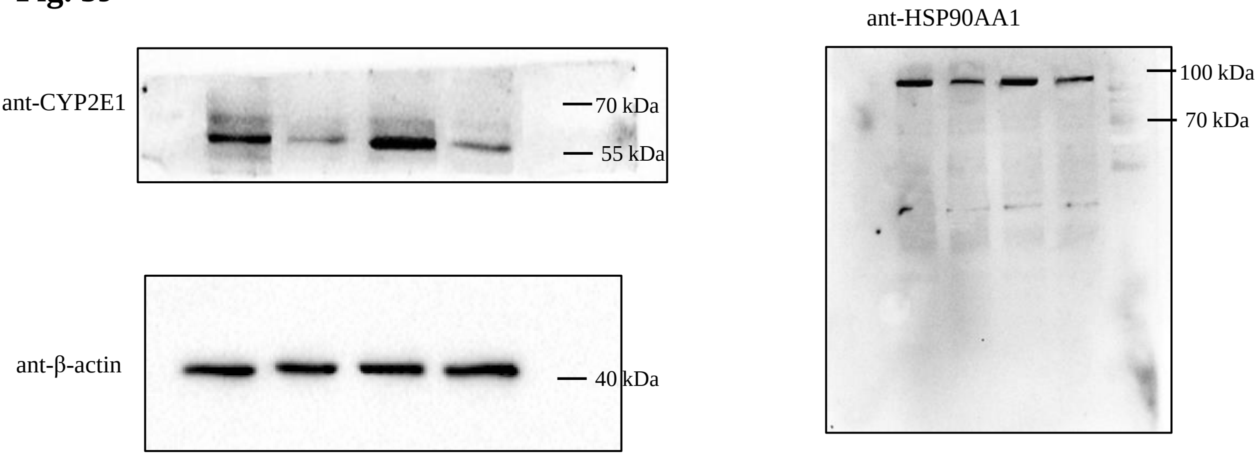

Fig. 6A

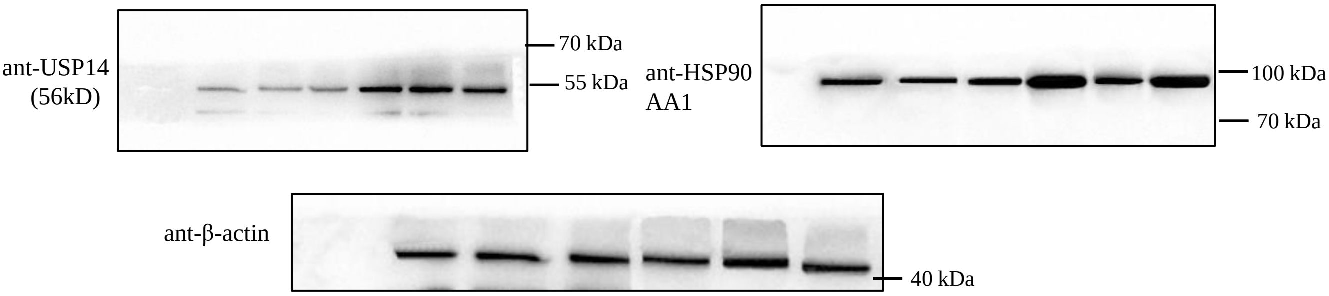

Fig. 6B

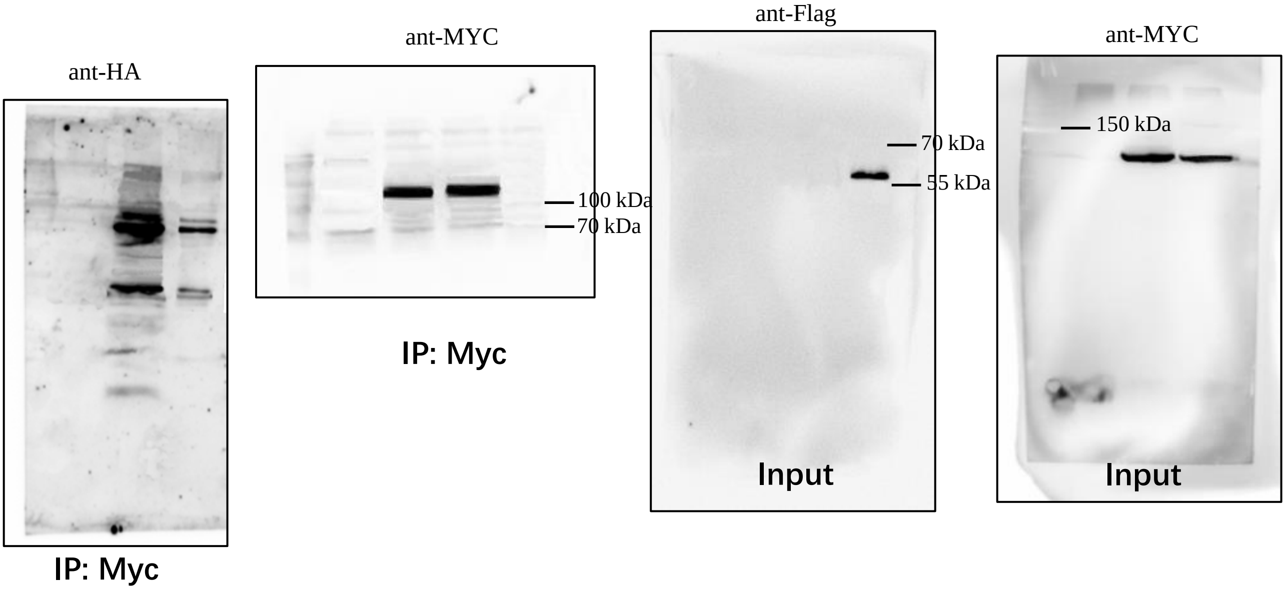

Fig. 6D

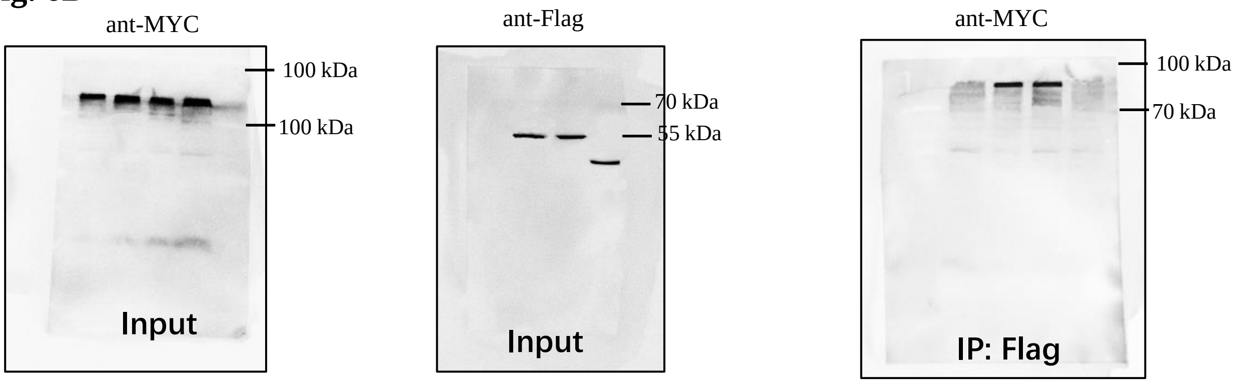

Fig. 6E

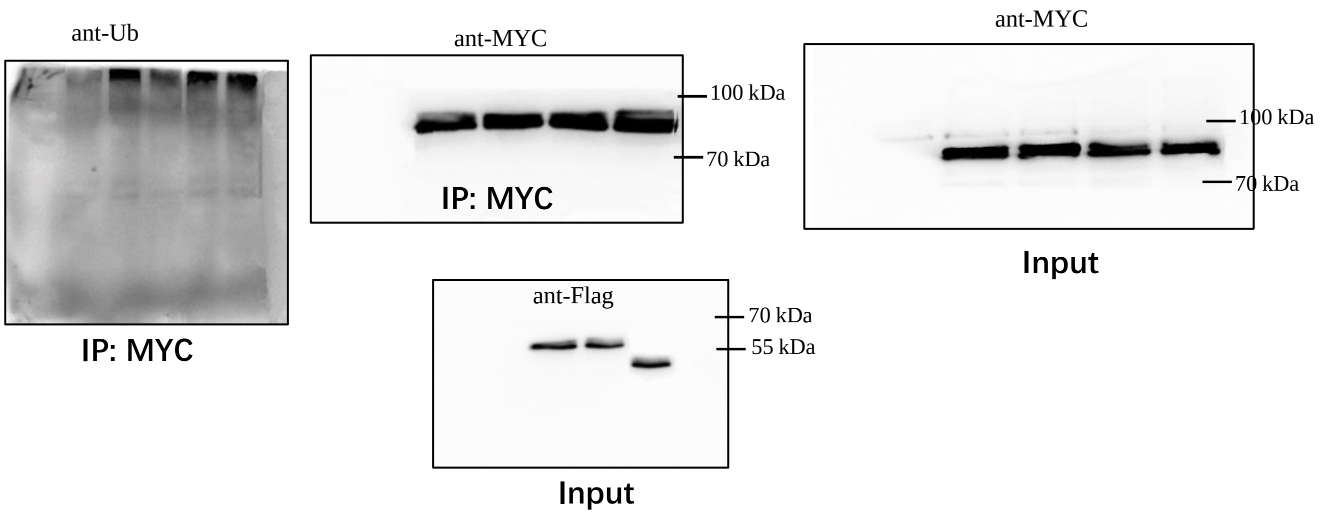

Fig. 6F

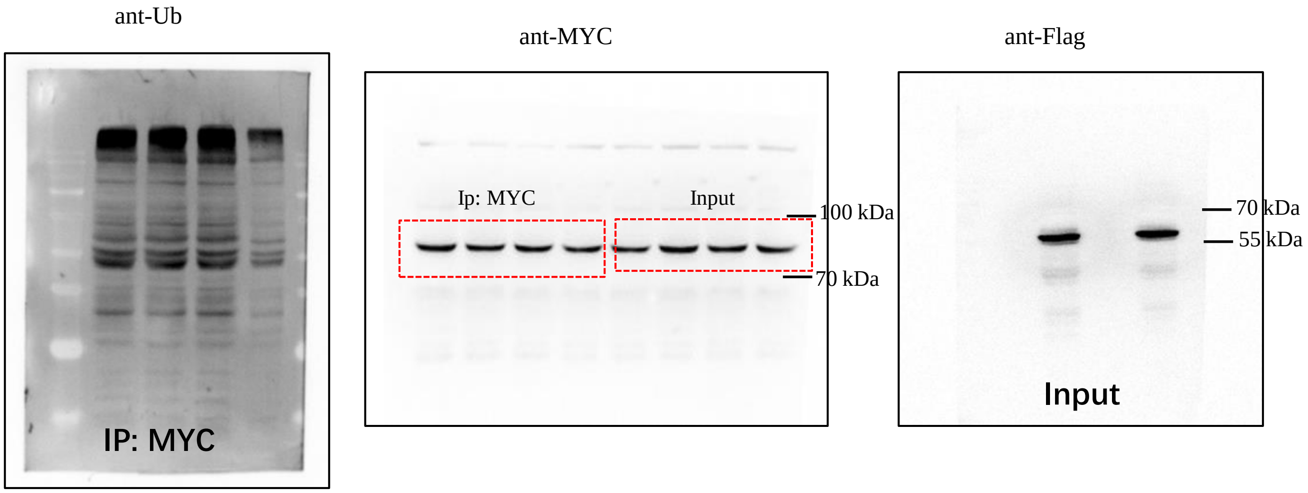

Fig. S6A

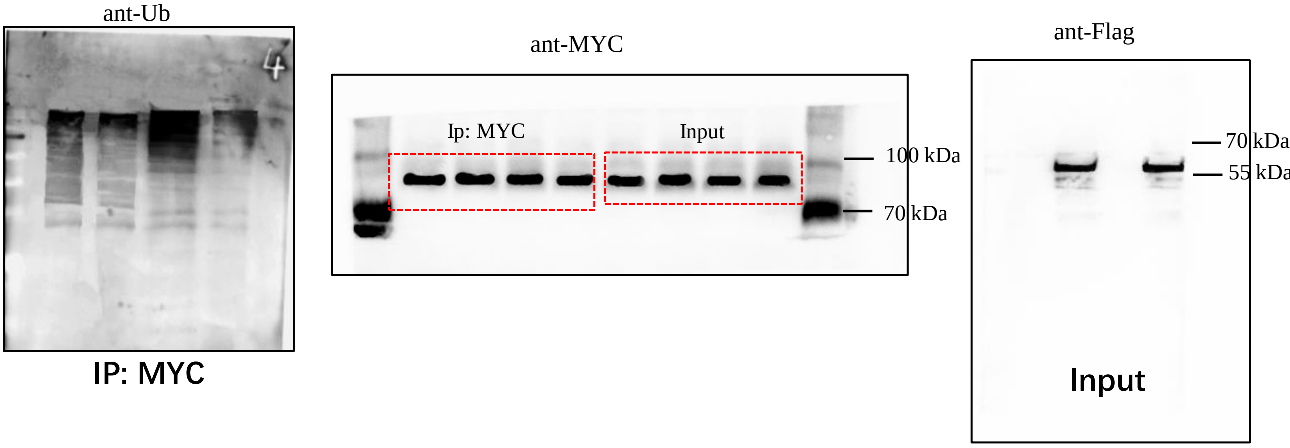

Fig. S6B

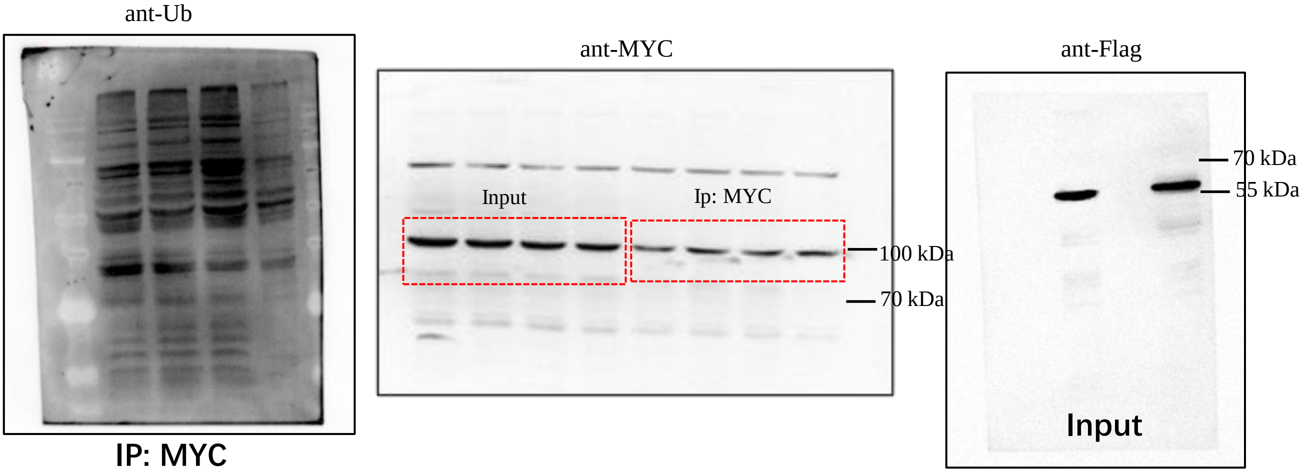

Fig. 6G

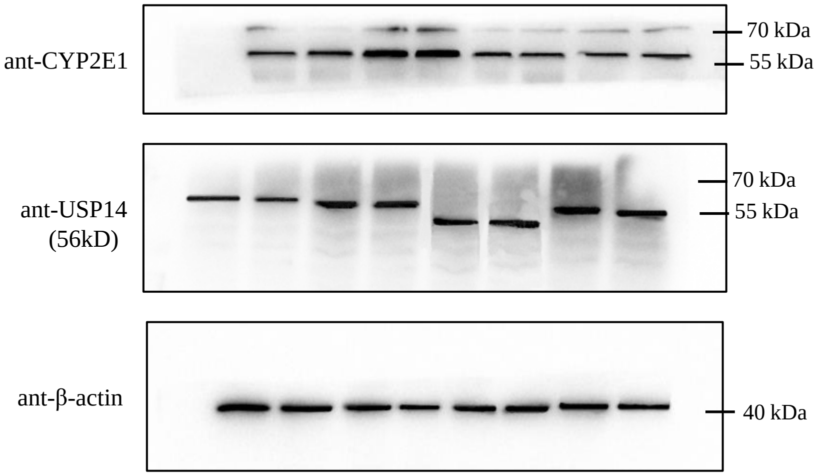

Fig. S7A

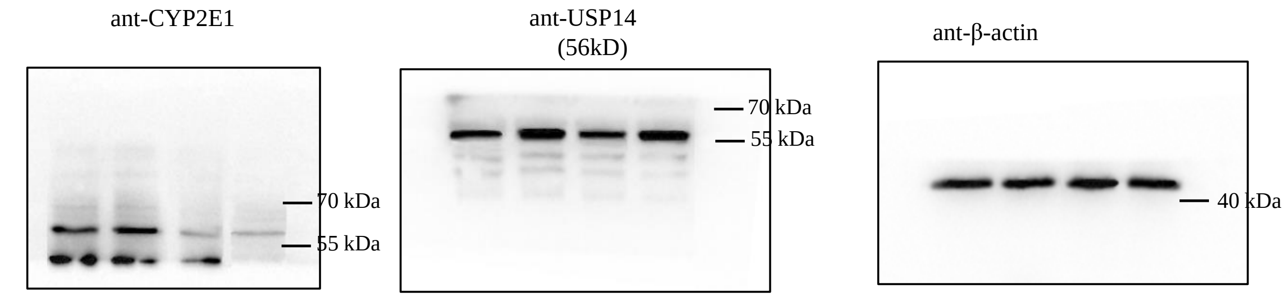

Fig. S7B

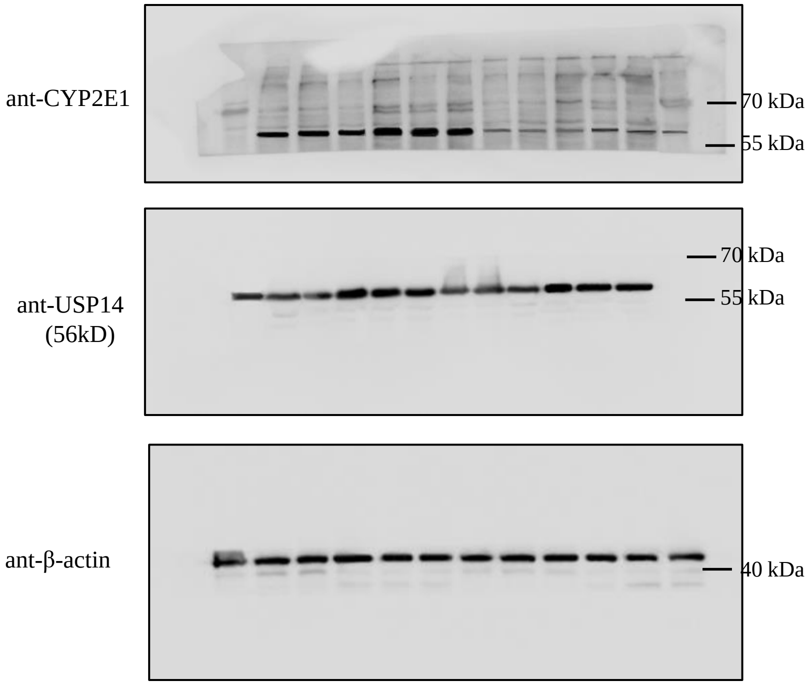

Supplement: Supplementary file 2 — Original Data File [file 41419_2023_6091_MOESM2_ESM.pdf]
